# Indoxyl sulfate induces intestinal barrier injury through IRF1-DRP1 axis-mediated mitophagy impairment

**DOI:** 10.7150/thno.45455

**Published:** 2020-06-05

**Authors:** Yinghui Huang, Jie Zhou, Shaobo Wang, Jiachuan Xiong, Yin Chen, Yong Liu, Tangli Xiao, Yi Li, Ting He, Yan Li, Xianjin Bi, Ke Yang, Wenhao Han, Yu Qiao, Yanli Yu, Jinghong Zhao

**Affiliations:** 1 Department of Nephrology, the Key Laboratory for the Prevention and Treatment of Chronic Kidney Disease of Chongqing, Kidney Center of PLA, Xinqiao Hospital, Army Medical University (Third Military Medical University), Chongqing, 400037, China.; 2 Institute of Combined Injury, State Key Laboratory of Trauma, Burns and Combined Injury, Chongqing Engineering Research Center for Nanomedicine, College of Preventive Medicine, Army Medical University, Chongqing 400038, China.; 3 Department of Oncology and Southwest Cancer Center, Southwest Hospital, Army Medical University, Chongqing 400038, China.

**Keywords:** chronic kidney disease, indoxyl sulfate, intestinal barrier injury, mitophagy, dynamin-related protein 1

## Abstract

**Rationale:** The dysfunctional gut-kidney axis forms a vicious circle, which eventually becomes a catalyst for the progression of chronic kidney disease (CKD) and occurrence of related complications. However, the pathogenic factors of CKD-associated intestinal dysfunction and its mechanism remain elusive.

**Methods:** We first identified the protein-bound uremic toxin indoxyl sulfate (IS) as a possible contributor to intestinal barrier injury. Transepithelial electrical resistance, permeability assay and transmission electron microscopy were carried out to evaluate the damaging effect of IS on intestinal barrier in intestinal epithelial cells, IS-injected mice and CKD mice. In vitro and in vivo experiments were performed to investigate the role of IS in intestinal barrier injury and the underlying mechanism. Finally, CKD mice treated with AST-120 (an oral adsorbent for IS) and gene knockout mice were used to verify the mechanism and to explore possible interventions for IS-induced intestinal barrier injury.

**Results:** Transepithelial electrical resistance and the expressions of tight junction-related genes were significantly suppressed by IS in intestinal epithelial cells. In vitro experiments demonstrated that IS inhibited the expression of dynamin-related protein 1 (DRP1) and mitophagic flux, whereas DRP1 overexpression attenuated IS-induced mitophagic inhibition and intestinal epithelial cell damage. Furthermore, IS suppressed DRP1 by upregulating the expression of interferon regulatory factor 1 (IRF1), and IRF1 could directly bind to the promoter region of DRP1. Additionally, the decreased expression of DRP1 and autophagosome-encapsulated mitochondria were observed in the intestinal tissues of CKD patients. Administration of AST-120 or genetic knockout of IRF1 attenuated IS-induced DRP1 reduction, mitophagic impairment and intestinal barrier injury in mice.

**Conclusions:** These findings suggest that reducing IS accumulation or targeting the IRF1-DRP1 axis may be a promising therapeutic strategy for alleviating CKD-associated intestinal dysfunction.

## Introduction

Chronic kidney disease (CKD) affects as much as 10-15% of the adult population, and accounts for approximately 1.5% of deaths worldwide 1-3. Intestinal dysfunction emerges as one of the most common complications of CKD, mainly clinically manifested by anorexia, vomiting, diarrhea, constipation, and mucosal hemorrhage and erosion 1, 2, 4. Conversely, intestinal dysfunction aggravates renal injury via gut microbiota, uremic toxins and systemic inflammation 5. Previous studies have mainly focused on the effect of intestinal dysfunction on CKD progression and occurrence of CKD complications. However, the mechanisms of another aspect of the gut-renal axis, CKD-associated intestinal dysfunction, lack in-depth exploration 6, 7. Recently, treatment intervention for intestinal dysfunction and the alteration of intestinal flora have been considered an effective avenue to prevent the progression of CKD and its complications 1, 2, 4, 5, 7-9. Therefore, investigating the pathogenesis of CKD-associated intestinal dysfunction would provide clinical implications for the development of novel therapeutic strategies targeting the gut-kidney axis 5.

Intestinal dysfunction is characterized by abnormally increased intestinal permeability, an indicator of impaired intestinal barrier 10, 11. The selectively permeable intestinal barrier surface was mainly constituted by a layer of intestinal epithelial cells (IECs), regulating the passage of ions and small molecules while protecting against the luminal antigens, toxins and pathogens 10-13. Thus, the damage of IECs leads to a marked loss of barrier function 11, 12. As reported, intestinal barrier properties are established by tight junction, adherens junction and desmosomes, among which tight junction is the most essential structure 10, 12. Defects in tight junction integrity are considered a crucial cause of increased permeability and intestinal dysfunction, while restoration of epithelial tight junction may improve the conditions 11-13. However, the role of tight junction impairment in CKD-associated intestinal dysfunction and related mechanisms are not fully understood.

Emerging evidence suggests that uremic toxin, a CKD-specific risk factor, was involved in intestinal barrier injury 14, 15. Advanced oxidation protein products (AOPPs) were demonstrated to induce IEC death through a redox-dependent pathway 14. An *in vitro* study found disruption of intestinal tight junction and barrier function by urea 15. Although a few studies have initially explored the effect of uremic toxins on intestinal barrier, their interaction with the gut microbiota and possible role in intestinal barrier injury are far from being elucidated. In particular, the role of protein-bound toxins in CKD-associated intestinal dysfunction should be probed deeply, as they are derived from utilization of amino acids by intestinal bacteria and difficult to be removed by routine dialysis. Therefore, in the present study, we focused on the contribution of a typical uremic protein-bound toxin indoxyl sulfate (IS) to intestinal barrier injury. Our findings demonstrate that IS induces intestinal barrier injury via inhibiting mitophagic flux of IECs. Furthermore, interferon regulatory factor 1 (IRF1)-mediated suppression of dynamin-related protein 1 (DRP1) contributes to IS-induced mitophagy inhibition.

## Results

### Intestinal barrier injury and dysbacteriosis were observed in CKD mice

**Figure 1:**
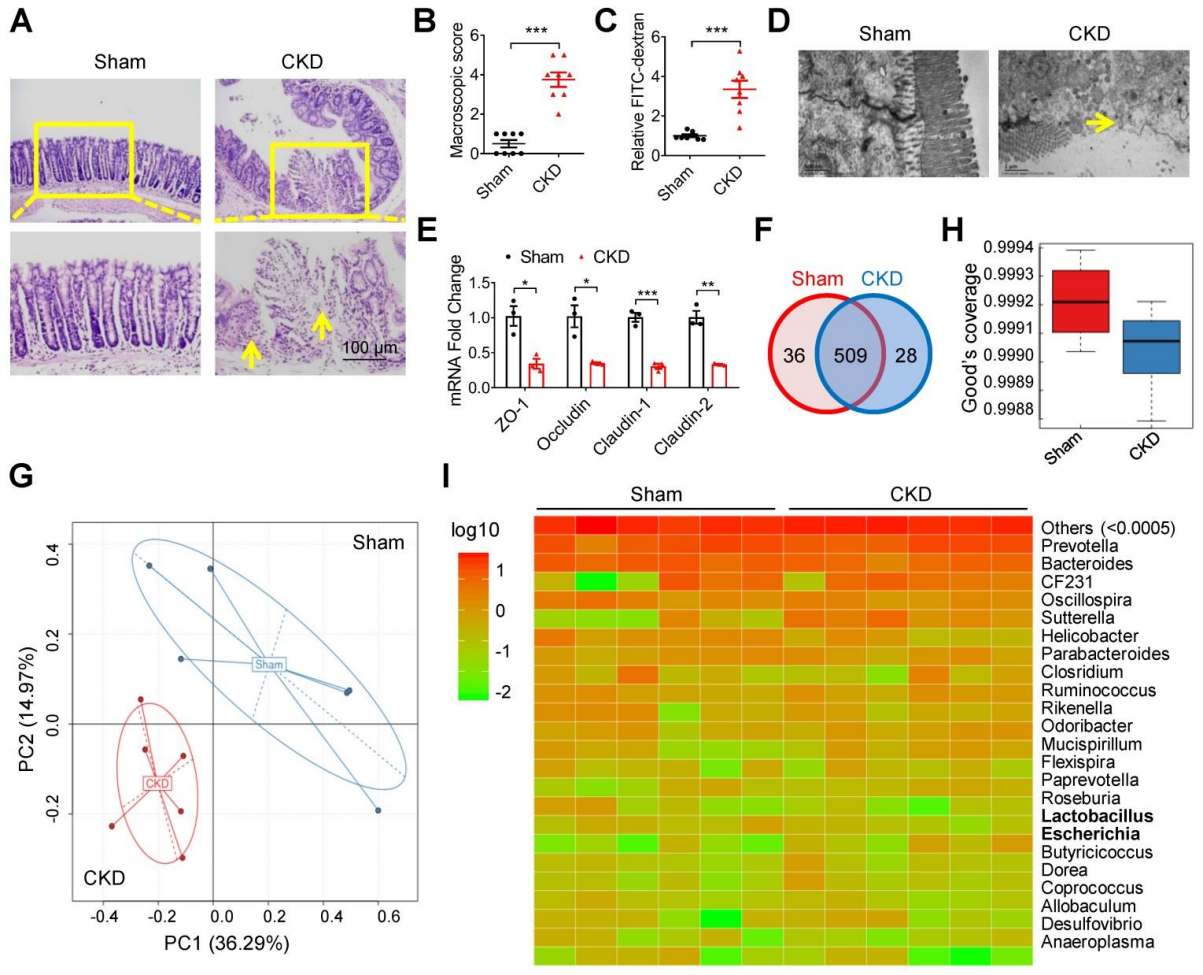
** Intestinal barrier injury and dysbacteriosis were observed in CKD mice. (A-E)** CKD mouse model was constructed, taking sham mice as control. n=8 per group. HE staining **(A)**, macroscopic injury score **(B**, detailed information was shown in Table S5**)**, intestinal permeability **(C)**, transmission electron microscopy (TEM) observation of tight junction **(D)** and qPCR analysis of tight junction-related genes **(E)** of intestinal tissues from sham and CKD mice. Yellow arrow indicates intestinal mucosal damage in **(A)** and impaired tight junction in **(D)**. **(F-I)** Fresh fecal samples of sham and CKD mice were collected for 16s ribosomal RNA (rRNA) sequencing and analysis. n=6 per group. Venn analysis **(F)**, principal component analysis** (G)**, alpha diversity comparison** (H)** and heatmap analysis **(I)** between sham and CKD mice. Data are shown as mean ± SEM and were analyzed by two-tailed unpaired Student's *t* test (**B, C, E**). * P<0.05, ** P<0.01, *** P<0.001.

In a 5/6 nephrectomy mouse model of CKD 16, goblet cells reduction, villi necrosis, edema and ulceration were observed in intestinal tissues (Figure 1A). The macroscopic injury score and intestinal permeability were much higher in CKD mice than in sham mice (Figure 1B and C). Notably, transmission electron microscopy (TEM) observation showed indistinct tight junction, reduced density and widened intercellular space in the intestinal tissues of CKD mice (Figure 1D). Meanwhile, the expressions of tight junction-related genes (zona occludens 1 (ZO-1), Occludin, Claudin-1 and Claudin-2) were also significantly decreased in CKD mice (Figure 1E). These results collectively suggest intestinal barrier injury in CKD mice. Since imbalance of gut flora contributes to intestinal barrier injury 6, 17, we carried out 16s ribosomal RNA (rRNA) sequencing. Venn analysis and Principal Component Analysis (PCA) revealed significant changes of Operational Taxonomic Unit (OUT) between sham and CKD mice (Figure 1F and G), and the alpha diversity comparison exhibited lower diversity in CKD mice (Figure 1H), indicating dysbacteriosis in CKD mice. Heatmap analysis verified multiple alterations of bacterial flora at species level, among which *Escherichia coli* (*E. coli*) population increased while *Lactobacillus* decreased (Figure 1I).

### Indoxyl sulfate induces intestinal epithelial cell injury *in vitro* and *in vivo*

Because indole is mainly produced by *E. coli* from tryptophan via tryptophanase, while *Lactobacillus* could competitively inhibit the production of indole through metabolizing tryptophan into indole-3-aldehyde 18-22, we first investigated whether indole could directly induce IEC injury. As a result, neither transepithelial electrical resistance (TER, the most sensitive measure of intestinal barrier), nor the expression of tight junction-related genes were repressed by indole in Caco2 cells, a colon epithelial cell line. Rather, indole enhanced TER and the expressions of Claudin-1 and Claudin-2 at a concentration of 1 mM (Figure S1A and B), indicating harmless influence of indole on IECs.

**Figure 2:**
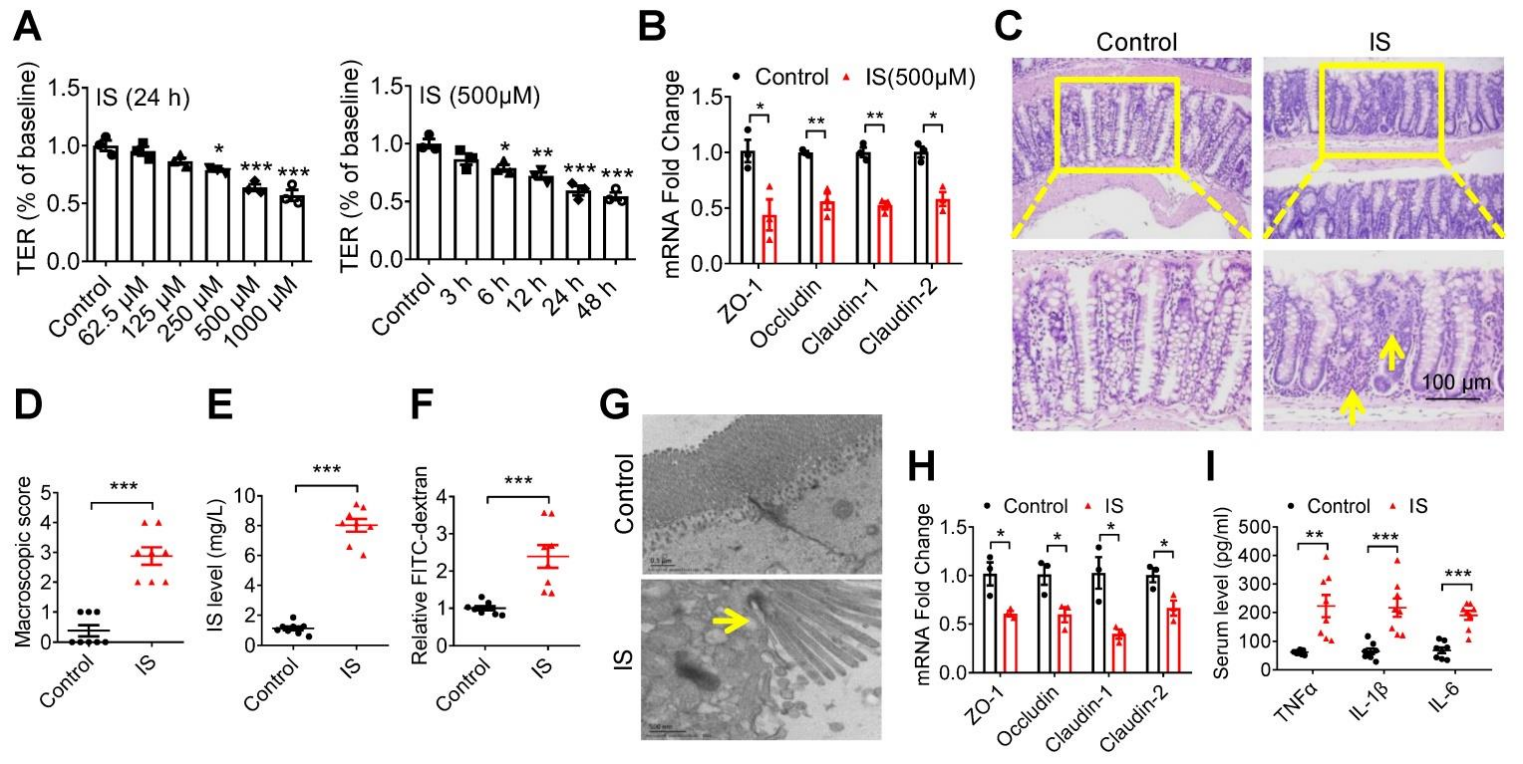
** Indoxyl sulfate (IS), a metabolite of intestinal flora, induces intestinal barrier injury. (A)** Caco2 cells were seeded on Millicell Hanging Cell Culture Inserts in 24-well plates and cultured for 14 days, and then treated with various concentrations of IS for 24 hours or 500 μM IS for different durations for TER determination. n=3. **(B)** qPCR analysis of the expression of tight junction-related genes in Caco2 cells treated with 500 μM IS for 24 hours. n=3. **(C-H)** Mice were intraperitoneally injected with IS (100 mg/kg) daily for 8 weeks. n = 8 per group. HE staining **(C)** and macroscopic injury score **(D**, detailed information was shown in Table S5**)** of intestinal tissues from control and IS-injected mice. **(E)** Serum IS level was determined using HPLC. Relative intestinal permeability **(F)**, TEM observation of tight junction **(G),** and qPCR analysis of tight junction-related genes **(H)** of intestinal tissues from mice in **(C)**. **(I)** Serum level of TNF-α, IL-1β and IL-6 from mice in **(C)** were measured using ELISA kits. Yellow arrow indicates intestinal mucosal damage in **(C)** and impaired tight junction in **(G)**. Data are shown as mean ± SEM and were analyzed by one-way ANOVA (**A**) or two-tailed unpaired Student's *t* test (**B, D-F, H, I**). * P<0.05, ** P<0.01, *** P<0.001.

Given this result, we further evaluated IS, an indole derivative accumulating in the blood with the progression of CKD 20. Then, we found that both TER and the expressions of tight junction-related genes were significantly suppressed by IS (Figure 2A and B). Further, increased intestinal macroscopic injury and permeability, accompanied by a higher level of serum IS, were observed in IS-injected mice (Figure 2C-F). Accordingly, impaired tight junction in epithelial tissues and increased serum inflammatory factors were detected in IS-injected mice (Figure 2G-I). These results collectively suggest that IS contributes to IEC injury.

### Indoxyl sulfate induces intestinal epithelial cell damage via inhibiting mitophagic flux

**Figure 3:**
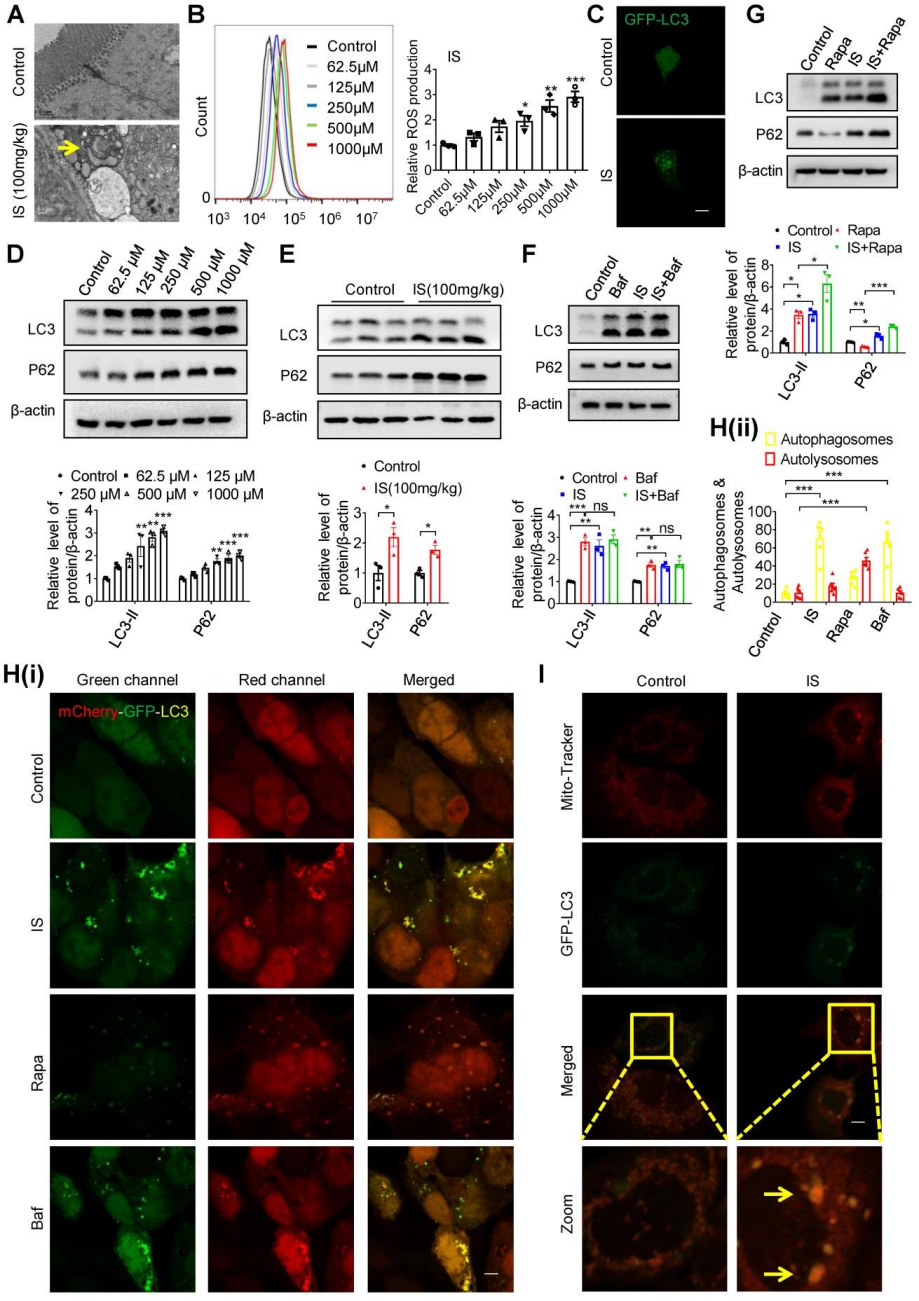
** IS induces intestinal damage via inhibiting mitophagic flux in Caco2 cells. (A)** TEM observation of autophagic vacuoles (indicated by yellow arrow) in intestinal tissues from control and IS-injected mice. **(B)** ROS production of Caco2 cells treated with various concentrations of IS for 24 hours. **(C)** GFP-LC3 plasmids were transfected into Caco2 cells using Lipofectamine 2000. Cells were treated with control or IS for another 24 hours for confocal microscopy observation. **(D)** Western blot analysis of LC3 and P62 expression of cells in **(B)**.** (E)** Western blot analysis of LC3 and P62 expression in intestinal tissues from control and IS-injected mice. **(F, G)** Western blot analysis of LC3 and P62 expression in Caco2 cells treated with control, Baf, IS or IS+Baf **(F)**; control, Rapa, IS or IS+Rapa **(G)**.** (H)** Caco2 cells were transfected with mCherry-GFP-LC3 adenovirus and treated with control, IS, Rapa or Baf for another 24 hours for confocal microscopy observation and ImageJ analysis.** (I)** Caco2 cells were transfected with GFP-LC3 plasmids, treated with control or IS for 24 hours and stained with mito-tracker for confocal microscopy. Yellow arrow indicates co-localization of mito-tracker and GFP-LC3 puncta. The gray scale of bands was quantified using ImageJ software. Scale bar, 10 μm. Data are shown as mean ± SEM and were analyzed by one-way ANOVA (**B, D, F-H**) or two-tailed unpaired Student's *t* test (**E**). n=3. ns: no significance. * P<0.05, ** P<0.01, *** P<0.001.

Interestingly, we observed increased autophagosome-encapsulated mitochondria in IECs in IS-injected mice by electron microscopy (Figure 3A). In fact, promoting ROS production is the main mechanism of various IS-induced cellular injuries 16, 23. Damaged mitochondria are important sources of increased ROS, and selective removal of these impaired organelles via mitophagy plays a pivotal role in maintaining cellular homeostasis 24. Therefore, we speculate that IS may induce intestinal epithelial damage by affecting mitophagy. To test this hypothesis, we incubated Caco2 cells with IS at various concentrations, and found an increased ROS production in a dose-dependent manner (Figure 3B). After transfection of green fluorescent protein (GFP)-LC3 plasmids into Caco2 cells, GFP-LC3 puncta were observed in IS-treated cells (Figure 3C). Since increased autophagic vacuoles (AVs) are attributed to enhanced autophagic flux or impaired AV clearance, we next distinguished between these two possibilities. Western blot analysis verified that both LC3-II and autophagy substrate P62 (also known as sequestosome 1, SQSTM1) were upregulated by IS in Caco2, HIEC, primary colon epithelial cells and intestinal epithelial tissues of mice (Figure 3D, 3E, S2), indicating a potential inhibitory effect of IS on autophagic flux. Meanwhile, IS-induced accumulation of LC3-II and P62 was not aggravated by Bafilomycin A1 (Baf, an autophagic degradation blocker), whereas Rapa (an autophagic inducer)-induced LC3-II expression was significantly enhanced by IS (Figure 3F and G). Furthermore, yellow dots were yielded in IS-treated cells transfected with mCherry-GFP-LC3 adenovirus (Figure 3H). These findings collectively reveal that IS is a potent autophagy inhibitor.

Synchronously, a decrease in ATP level and loss of mitochondrial membrane potential were detected in Caco2 cells (Figure S3A-C), indicating that IS induced mitochondrial dysfunction. Co-localization of mito-tracker and GFP-LC3 puncta, elevated level of mitochondrial DNA (mtDNA) copy number and the increased expression of cytochrome c oxidase subunit IV (COXIV, a mitochondrial marker) were observed in IS-treated IECs (Figure 3I, S3D-E). As mitochondrial number can be regulated by biogenesis or mitophagy-mediated removal 25, we also detected the expressions of two biomarkers of mitochondrial biogenesis, peroxisome proliferator-activated receptor gamma coactivator 1 alpha (PGC1α) and mitochondrial transcription factor A (TFAM), and found no effect of IS on their expressions (Figure S3E). These results suggest that IS might induce IEC injury through inhibiting mitophagy.

### Indoxyl sulfate inhibits mitophagy through suppressing DRP1 in intestinal epithelial cells

**Figure 4:**
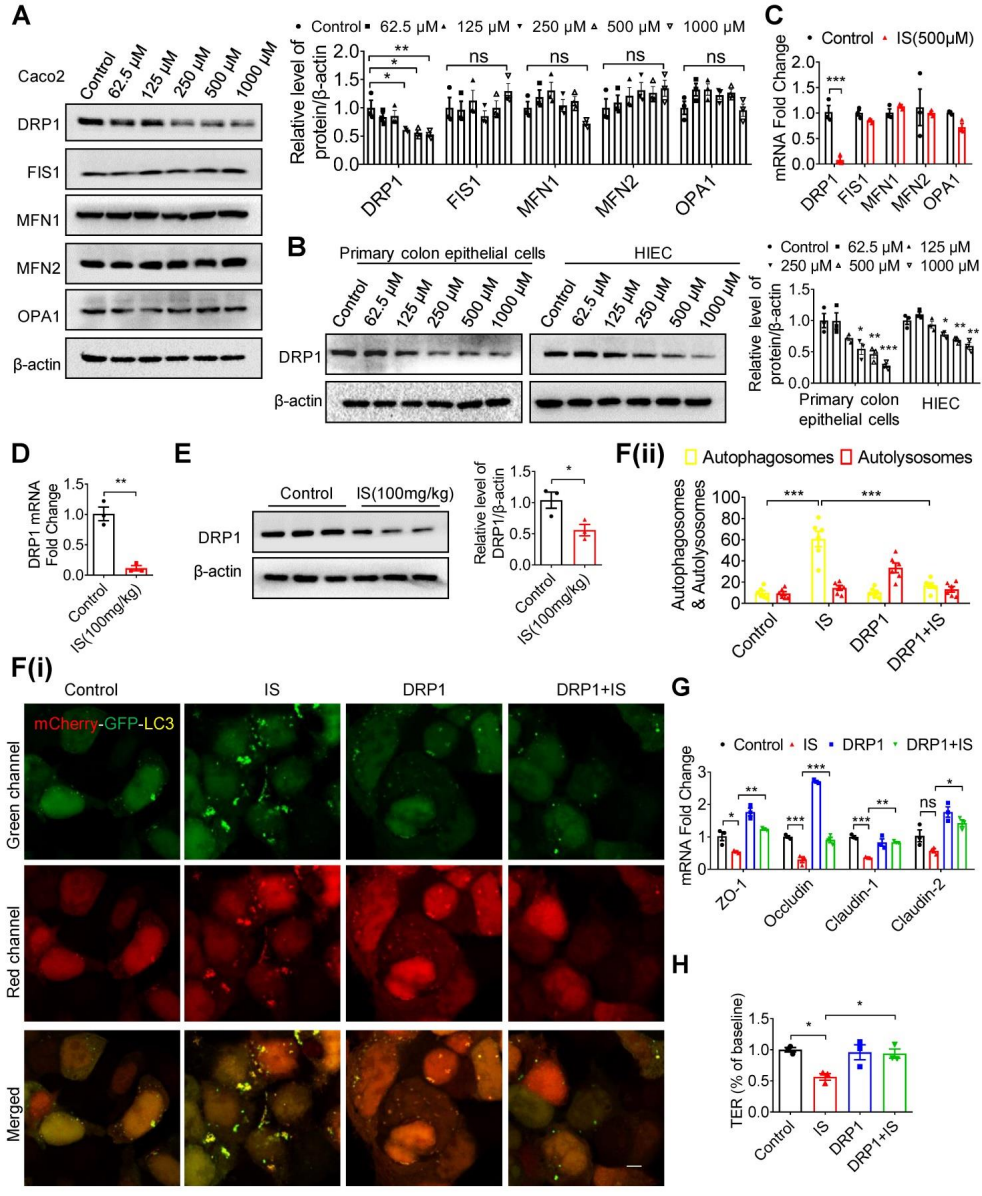
** IS inhibits mitophagy and intestinal function through suppressing DRP1. (A-C)** Western blot and qPCR analysis of mitochondrial fission and fusion-related proteins in Caco2 **(A, C)**, HIEC and primary colon epithelia cells **(B)** treated with control or IS for 24 hours. **(D, E)** qPCR **(D)** and Western blot **(E)** analysis of DRP1 expression in intestinal tissues from control or IS-injected mice. **(F)** Caco2 cells were transfected with mCherry-GFP-LC3 in combination with DRP1 overexpression plasmids or vector control, and then treated with control or IS for 24 hours for confocal microscopy and ImageJ analysis. Scale bar, 10 μm. **(G)** Caco2 cells were transfected with DRP1 overexpression plasmids or vector control and then treated with control or IS for 24 hours. The expressions of tight junction-related genes were detected using qPCR analysis. **(H)** Caco2 cells were seeded on Millicell Hanging Cell Culture Inserts in 24-well plates and cultured for 14 days, and then transfected with DRP1 overexpression plasmids or vector control and treated with control or IS for 24 hours for TER determination. The gray scale of bands was quantified using ImageJ software. Data are shown as mean ± SEM and were analyzed by one-way ANOVA (**A, B, F-H**) or two-tailed unpaired Student's *t* test (**C-E**). n=3. ns: no significance. * P<0.05, ** P<0.01, *** P<0.001.

To investigate the mechanism by which IS inhibited mitophagy, the classical mammalian target of rapamycin (mTOR) signaling pathway was firstly examined in Caco2 cells, but no changes were found (Figure S4A). As reported, the impairment of dynamic balance of mitochondrial fusion and fission leads to impaired mitophagy and mitochondrial function 26. Therefore, we screened the classical genes and demonstrated that DRP1 expression was significantly inhibited by IS in IECs and intestinal tissues, while the expressions of fission 1 (FIS1), mitofusin 1 (MFN1), mitofusin 2 (MFN2) and optic atrophy 1 (OPA1) were not regulated by IS (Figure 4A-E). Concordantly, confocal microscopy showed elongated mitochondria in IS-treated cells (Figure S4B), which coincided with the mitochondrial fission-promoting effect of DRP1. To further investigate the role of DRP1, we treated Caco2 cells with Mdivi1, a classic DRP1 inhibitor, in the presence or absence of IS. Mdivi1 significantly aggravated IS-induced ROS production and autophagy inhibition (Figure S5A-C). On the other hand, DRP1 overexpression plasmids were transfected into Caco2 cells. The overexpressed DRP1 restrained IS-induced ROS production and, like NAC (a ROS scavenger), abated autophagy inhibition (Figure S5D-G). Moreover, DRP1 overexpression significantly attenuated IS-induced mitophagy inhibition and tight junction impairment in Caco2 cells (Figure 4F-H). These results hint that IS induces mitophagic impairment and IEC injury via repressing DRP1.

### Indoxyl sulfate inhibits DRP1 through enhancing IRF1 expression

**Figure 5:**
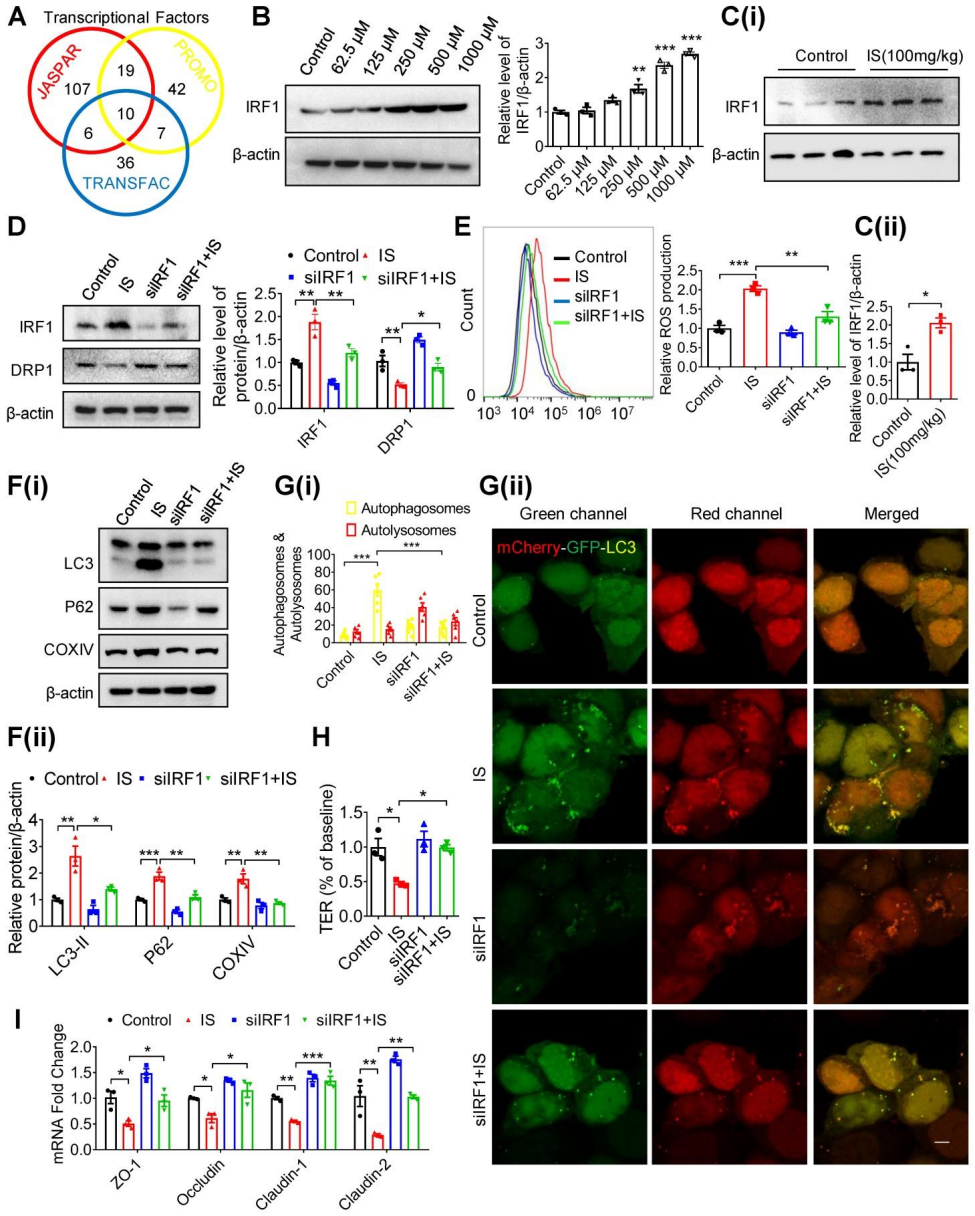
** IS inhibits DRP1 through enhancing IRF1 expression. (A)** Transcriptional factors in DRP1 gene promoter region were predicted using JASPAR, PROMO (ALGGEN) and TRANSFAC (gene-regulation) software programs, and 10 common transcriptional factors were found. **(B, C)** Western blot analysis of IRF1 expression in Caco2 cells **(B)** and intestinal tissues from mice **(C)** treated with control or IS. **(D-F)** Caco2 cells were transfected with siControl or siIRF1, and then treated with control or IS for 24 hours. Cells were harvested for Western blot analysis to detect the expressions of IRF1 and DRP1 **(D)**, flow cytometry to detect ROS production **(E)**, Western blot analysis to test for LC3, P62 and COXIV expression **(F)**. **(G)** Caco2 cells were transfected with mCherry-GFP-LC3 in combination with siIRF1 or control siRNA, and then treated with control or IS for 24 hours for confocal microscopy and ImageJ analysis. Scale bar, 10 μm. **(H-I)** TER determination **(H)** and qPCR analysis of tight junction-related genes **(I)** of Caco2 cells transfected with siControl or siIRF1, and then treated with control or IS for 24 hours. The gray scale of bands was quantified using ImageJ software. Data are shown as mean ± SEM and were analyzed by one-way ANOVA (**B, D-I**) or two-tailed unpaired Student's *t* test (**C**). n=3. * P<0.05, ** P<0.01, *** P<0.001.

To explore the upstream mediator of DRP1 in IS-regulated mitophagy, transcriptional factors with potential binding sites in the promoter region of the DRP1 gene were predicted using bioinformatics (Figure 5A). Subsequent qPCR screening demonstrated that Yin-Yang 1 (YY1) and IRF1 were upregulated by IS both *in vitro* and *in vivo* (Figure S6A and B). However, siRNA against YY1 (siYY1) did not rescue IS-induced downregulation of DRP1 (Figure S6C and D). Additionally, IRF1 protein was induced by IS in IECs and intestinal tissues (Figure 5B and C). DRP1 expression in Caco2 cells was downregulated by IRF1 overexpression (pIRF1) and upregulated by IRF1 knockdown (siIRF1) (Figure S6E and F). *In vitro*, knockdown of IRF1 significantly reversed IS-induced DRP1 repression, ROS production elevation, mitophagy inhibition, TER suppression and downregulation of tight junction-related genes (Figure 5D-I, S6G). These findings indicate that IS inhibits DRP1 by upregulating IRF1 expression.

**Figure 6:**
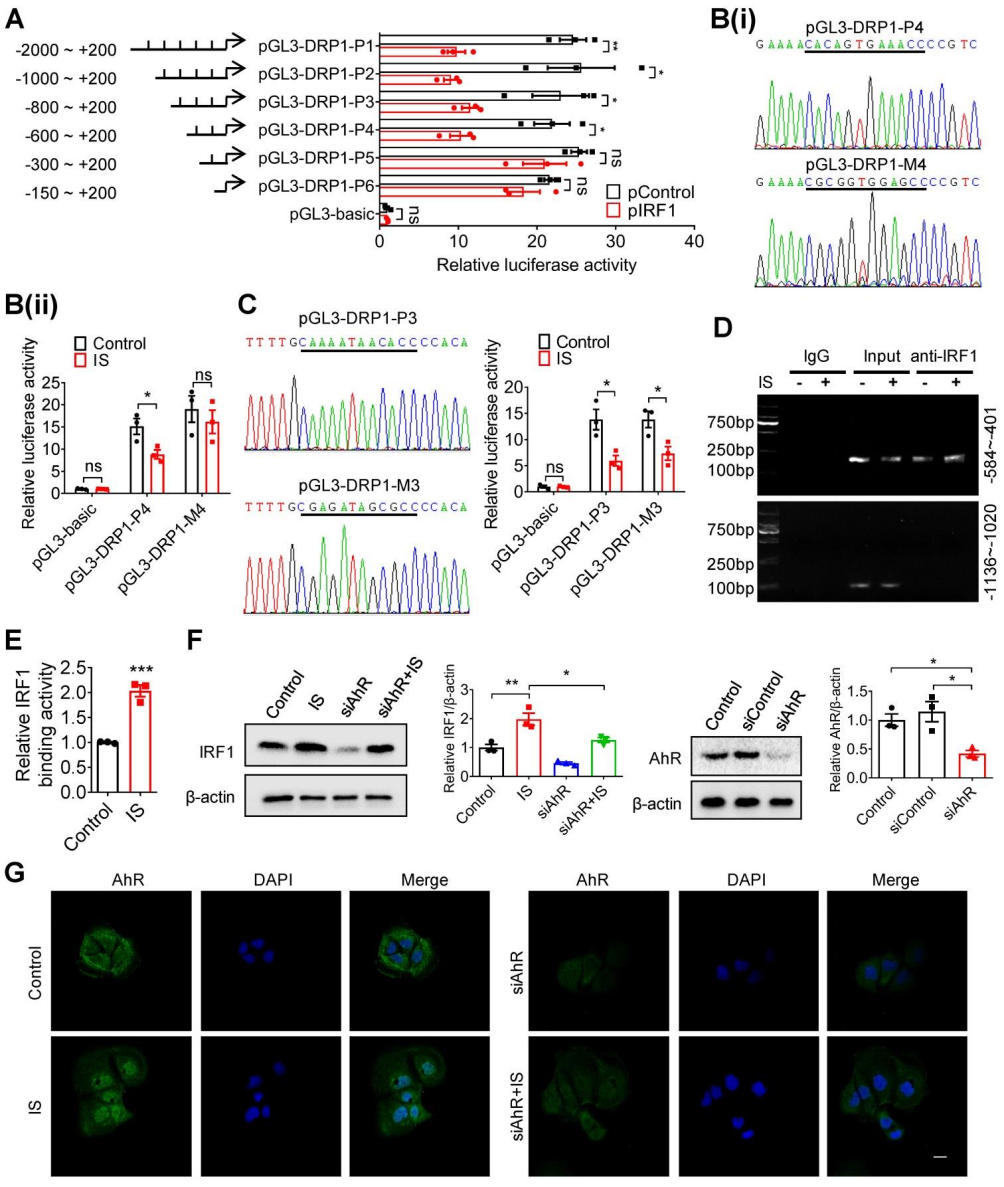
** IRF1 suppresses DRP1 transcription through directly binding to its promoter region. (A)** Caco2 cells were co-transfected with pRL-TK vector and pGL3-basic or recombinant plasmids containing various DRP1 promoter regions, in combination with pControl or pIRF1. Then, cells were harvested for dual-luciferase reporter assay. Firefly luciferase activity was normalized against Renilla activity. **(B, C)** Cells were co-transfected with pRL-TK and pGL3-basic, pGL3-DPR1-P4, pGL3-DPR1-M4, pGL3-DPR1-P3 or pGL3-DPR1-M3 (the mutated IRF1 binding sites were underlined) using Lipofectamine 2000, and treated with control or IS for another 24 hours for dual-luciferase reporter assay. **(D)** Cells were treated with control or IS for 24 hours and collected for ChIP assay. DNA was amplified using PCR primers (-584 ~ -401 and -1136 ~ -1020). **(E)** qPCR analysis of IRF1 antibody-immunoprecipitated DNA in **(D)**. **(F, G)** Cells were transfected with siAhR or siControl and treated with control or IS for another 24 hours. Cells were harvested for Western blot analysis to detect AhR and IRF1 expression **(F)** and for immunofluorescence confocal microscopy to evaluate the cellular localization of AhR **(G)**. Scale bar, 10 μm. The gray scale of bands was quantified using ImageJ software. Data are shown as mean ± SEM and were analyzed by two-tailed unpaired Student's *t* test (**A-C, E**) or one-way ANOVA (**F**). n=3. ns: no significance. * P<0.05, ** P<0.01, *** P<0.001.

Based on the predicted binding sites of IRF1 in DRP1 promoter region (Table S1), we hypothesized that IRF1 could inhibit DRP1 expression via directly binding to its promoter region. As shown in Figure 6A, pIRF1 significantly suppressed the luciferase activity of the recombinant luciferase reporters pGL3-DRP1-P1, pGL3-DRP1-P2, pGL3-DRP1-P3 and pGL3-DRP1-P4, while it had no effect on pGL3-DRP1-P5 or pGL3-DRP1-P6, implying an IRF1 response element within the sequence containing -600 to -300 nucleotides relative to the transcriptional start site. According to the bioinformatic prediction (Table S1), the binding sites should be the sequence containing -508 to -497 (CACAGTGAAACC). As expected, mutation of pGL3-DRP1-P4 (-508 ~ -497) abolished IS-regulated DRP1 promoter activity (Figure 6B), while mutation of pGL3-DRP1-P3 (-728 ~ -717) did not (Figure 6C). Further, ChIP assay was performed and revealed direct binding of IRF1 to DRP1 promoter region (-584 ~ -401), which could be enhanced by IS (Figure 6D and E).

As reported, IRF1 was the downstream gene of aryl hydrocarbon receptor (AhR), a canonical receptor of IS 27, 28. Further study demonstrated that siRNA against AhR (siAhR) significantly prevented IS-induced IRF1 upregulation (Figure 6F and G), suggesting that AhR contributes to IS-induced IRF1 upregulation.

### Intestinal barrier injury, disruption of the IRF1-DRP1 axis and mitophagic impairment were observed in intestinal tissues of CKD patients

**Figure 7:**
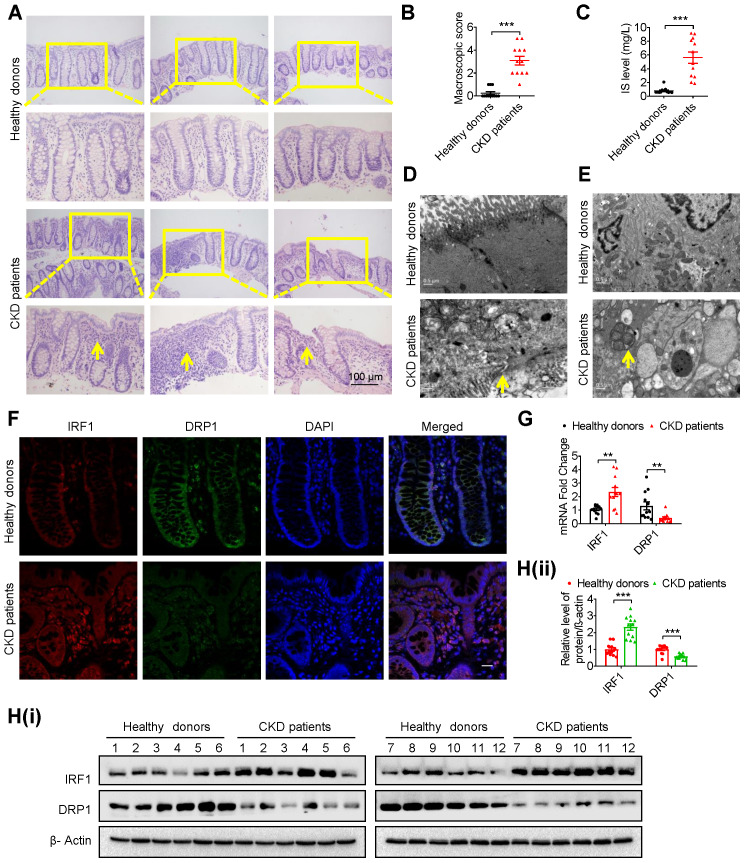
** Intestinal barrier injury, disruption of the IRF1-DRP1 axis and mitophagic impairment were observed in intestinal tissues of CKD patients. (A, B)** HE staining **(A)** and macroscopic injury score **(B**, detailed information was shown in Table S5**)** of intestinal tissues from healthy donors and CKD patients at stage 5. n=12 per group. Scale bar, 100 μm. **(C)** Serum IS level of (**A**) was determined using HPLC. **(D, E)** TEM observation of tight junction (**D**) and mitophagy (**E**) in intestinal tissues from (**A**). **(F-H)** The expressions of IRF1 and DRP1 in intestinal tissues from (**A**) were detected using immunofluorescence (**F**), qPCR (**G**) and Western blot analysis (**H**). Scale bar, 10 μm. The gray scale of bands was quantified with ImageJ software. Yellow arrow indicates intestinal mucosal damage in **(A)**, impaired tight junction in **(D)** and mitophagic vacuoles in **(E)**. Data are shown as mean ± SEM and were analyzed by two-tailed unpaired Student's *t* test (**B, C, G, H**). ** P<0.01, *** P<0.001.

To verify the above possible mechanisms and phenomena, the intestinal tissues of 12 CKD patients and 12 healthy donors were collected. Hematoxylin eosin (HE) staining showed goblet cell reduction, villi necrosis, ulceration and fibroplasia in intestinal tissues of CKD patients (Figure 7A and B). Correspondingly, a higher serum IS level, impaired tight junction and autophagosome-encapsulated mitochondria were observed in CKD patients (Figure 7C-E). Of note, immunofluorescence, qPCR and Western blot analysis confirmed increased IRF1 and decreased DRP1 expression in intestinal epithelial tissues of CKD patients (Figure 7F-H).

### Treatment with AST-120 or genetic knockout of IRF1 protects against indoxyl sulfate-induced DRP1 reduction, mitophagic impairment and intestinal barrier injury

**Figure 8:**
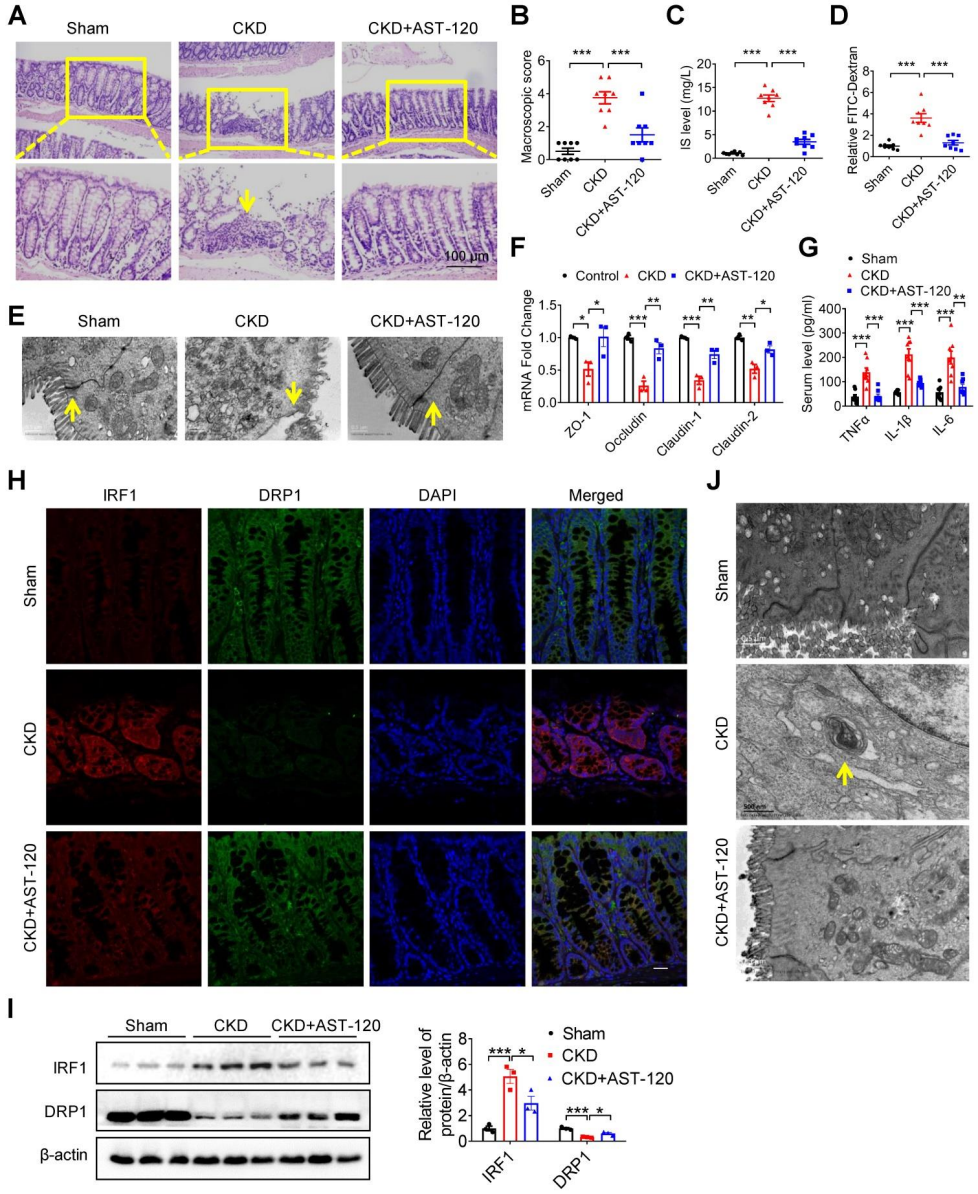
** Reducing indoxyl sulfate accumulation with AST-120 alleviated DRP1 reduction, mitophagic impairment and intestinal barrier injury in CKD mice. (A-H)** CKD mice were treated with or without AST-120. n=8 per group.** (A, B)** HE staining **(A)** and macroscopic injury score **(B**, detailed information was shown in Table S5**)** of intestinal tissues from sham, CKD and AST-120-treated CKD mice. **(C)** Serum IS level was analyzed using HPLC.** (D-F)** Relative intestinal permeability **(D)**, TEM observation of tight junction **(E)** and qPCR analysis of tight junction-related genes **(F)** of intestinal tissues from mice in **(A)**. **(G)** Serum levels of TNF-α, IL-1β and IL-6 in mice in **(A)** were detected using ELISA kits. **(H, I)** Immunofluorescence and Western blot analysis of the expressions of IRF1 and DRP1 in intestinal tissues from mice in **(A)**. Scale bar, 10 µm. **(J)** TEM observation of mitophagy in intestinal tissues from mice in **(A)**. Yellow arrow indicates intestinal mucosal damage in **(A)**, impaired tight junction in **(E)** and mitophagic vacuoles in **(J)**. Data are shown as mean ± SEM and were analyzed by one-way ANOVA (**B-D, F, G, I**). * P<0.05, ** P<0.01, *** P<0.001.

**Figure 9:**
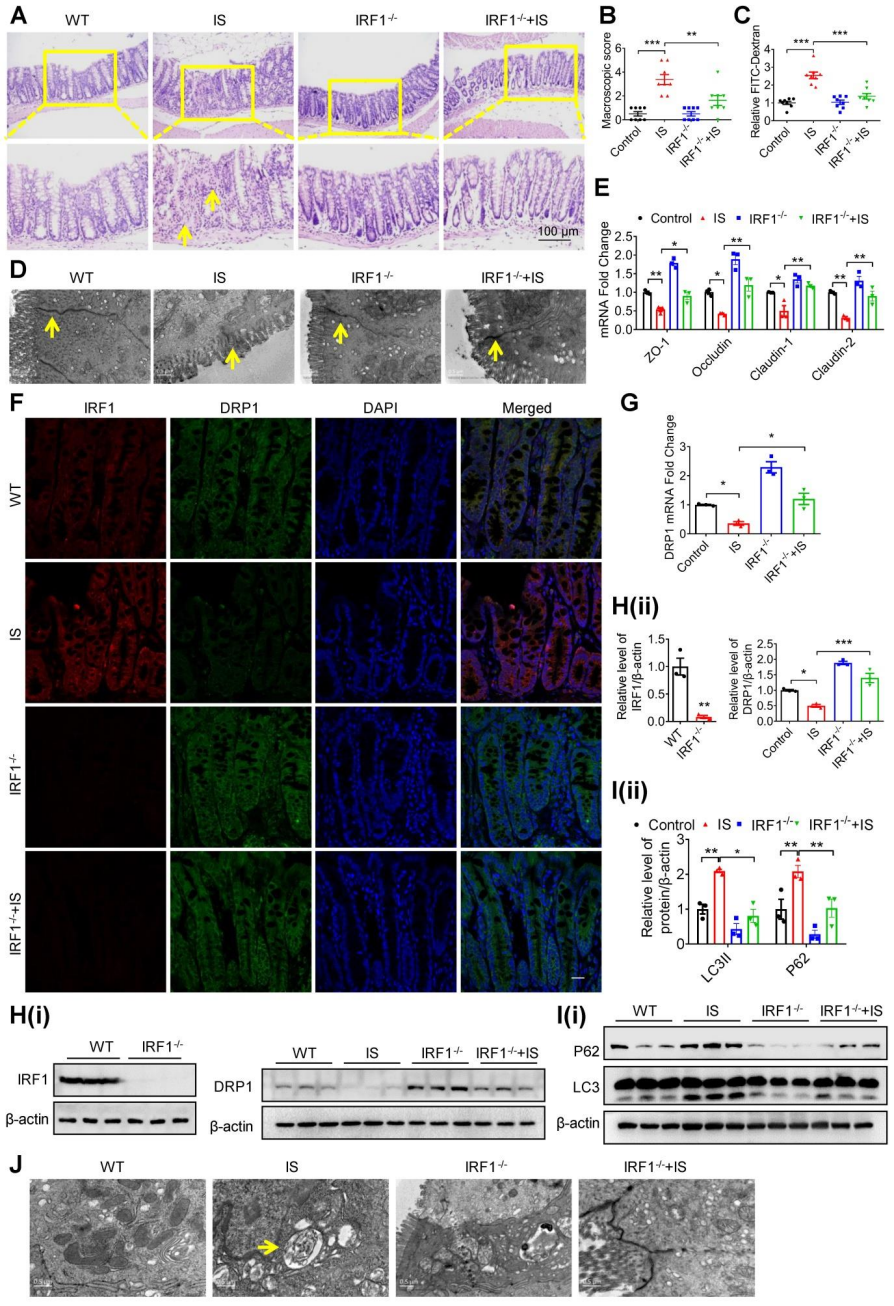
** Genetic knockout of IRF1 protects against IS-induced DRP1 reduction, mitophagic impairment and intestinal barrier injury. (A-E)** Wild type (WT) C57BL/6J and IRF1^-/-^ mice were treated with control or IS for 8 weeks. n=8 per group. HE staining **(A)**, macroscopic injury score **(B**, detailed information was shown in Table S5**)**, relative intestinal permeability **(C)**, TEM observation of tight junction **(D)** and qPCR analysis of tight junction-related genes **(E)** of intestinal tissues from control and IS-injected WT and IRF1^-/-^ mice. **(F)** The expressions of IRF1 and DRP1 were determined using immunofluorescence in intestinal tissues from mice in **(A)**. Scale bar, 10 μm. **(G-J)** qPCR and Western blot analysis of DRP1 expression **(G, H)**, Western blot analysis of P62 and LC3 expression **(I)**, and TEM observation of autophagy **(J)** in intestinal tissues from mice in **(A)**. The gray scale of bands was quantified using ImageJ software. Yellow arrow indicates intestinal mucosal damage in **(A)**, impaired tight junction in **(D)** and autophagic vacuoles in **(J)**. Data are shown as mean ± SEM and were analyzed by one-way ANOVA (**B, C, E, G, H** (right panel),** I**) or two-tailed unpaired Student's *t* test (**H**, left panel). ** P<0.01, *** P<0.001.

To further confirm the important role of IS and the IRF1-DRP1 axis in CKD-associated intestinal barrier injury, CKD mice were fed with AST-120, a charcoal adsorbent suppressing serum IS concentration as previously described 22. As anticipated, intestinal barrier injury, high serum IS level, decreased DRP1 expression and mitophagy blockade in CKD mice were significantly rescued by AST-120 (Figure 8). Moreover, IS-induced intestinal barrier injury and DRP1 downregulation were significantly alleviated in IRF1^-/-^ mice compared with wild type (WT) mice (Figure 9A-H). IS-mediated mitophagy inhibition was also restored in IRF1^-/-^ mice (Figure 9I and J). These findings collectively suggest that IS level reduction by AST-120 oral administration in CKD mice or IRF1 gene knockout (KO) in IS-injected mice attenuates DRP1 repression, mitophagic impairment in IECs and intestinal barrier injury.

## Discussion

Intestinal dysfunction emerges as a common complication of CKD patients, especially for those with end-stage renal disease (ESRD) 2, 4. However, there is still a lack of deep understanding of the pathogenesis of CKD-associated intestinal dysfunction. In the present study, we demonstrate that the uremic solute IS induces IEC injury via interfering mitophagic flux, in which process IS inhibits DRP1 expression by upregulating IRF1 expression. Accordingly, IRF1-DRP1 axis-mediated mitophagic inhibition was observed in CKD mice and patients. Oral administration of adsorbent AST-120 or genetic KO of IRF1 could attenuate IS-induced DRP1 reduction, mitophagic impairment and intestinal barrier injury in mice.

Gut dysbiosis is involved in CKD progression and increasingly recognized as a potential therapeutic target for CKD patients, but the mechanism of intestinal dysfunction in uremic milieus has not been fully elucidated 5, 7-9, 17, 20, 21, 29. In this study, we carried out 16s rRNA sequencing and identified an increase of *E. coli* population and a decrease of *Lactobacillus* species. Although different sequencing methods were used, previous reports consistently found decreased *Lactobacillus* and increased *Enterobacteriaceae* 17. Considering that *E. coli* produces indole and *Lactobacillus* inhibits indole production 18, 19, 30, we examined the relationship between indole and IEC injury but discovered that indole did not contribute to IEC injury, in agreement with a previous report 18. Therefore, we tested an indole derivative, IS, and found that IS was able to induce IEC injury *in vitro* and *in vivo*. Furthermore, reducing IS accumulation with the oral adsorbent AST-120 alleviated intestinal barrier injury in CKD mice. Recent studies identified that IS as a potential mediator of gut-kidney-heart/vessel crosstalk, and its increase paralleled with renal dysfunction and cardiovascular events 6, 7, 16, 17. Our study provides evidence that that gut-derived protein-binding toxin IS plays an important role in the vicious cycle of dysfunctional gut-kidney axis.

It is noteworthy that autophagic vacuoles were observed in intestinal epithelial tissues of IS-injected mice, while mitochondrial function of IECs was impaired by IS. Meanwhile, mitophagy was proved to protect vital organs by eliminating damaged mitochondria, including kidney and colon 24, 31-33. As reported, mitophagy was responsible for amelioration of inflammatory bowel disease (IBD) and dextran sulfate sodium (DSS)-induced colitis 24, 34. However, it is unclear whether mitophagy is involved in CKD-associated intestinal dysfunction. Previous studies (including ours) indicate that IS induces production of ROS in a variety of cells, mainly resulting from mitochondria and leading to mitochondrial damage in return 16, 20, 23. An *in vitro* study has found that IS induces ROS production and impairs the intactness of the IEC monolayer 35. Thus, mitophagy impairment may be involved in IS-induced intestinal dysfunction.

In this study, despite that the presence of mitophagic vacuoles in CKD patients did not mean the direct effect of indoxyl sulfate, mitophagic flux was significantly inhibited by IS *in vitro* and *in vivo*. However, canonical autophagy signaling pathway, mTOR, was not affected by IS. Reportedly, mitochondria are highly plastic organelles that constantly change their shape, structure and function through fusion and fission, known as mitochondrial dynamics 36. An excess of mitochondrial fission contributes to mitochondrial fragmentation, whereas enhanced mitochondrial fusion leads to mitochondrial hyper-tubulation, both of which ultimately result in mitochondrial dysfunction and mitophagic impairment 26, 37. This toxic effect could be restored to normal levels after simultaneous loss of fission and fusion-related genes, indicating that a balance of mitochondrial fission/fusion is fundamental to the maintenance of health 38.

A pervious report revealed that mitochondrial dynamics shifted to fusion in the kidneys of CKD rats 39, indicating disturbed mitochondrial dynamics in the uremic milieu. Therefore, we screened the expressions of mitochondrial dynamics-related genes and found that DRP1 expression was suppressed in IS-treated IECs, and this was also confirmed in intestinal tissues of IS-treated mice and CKD patients. DRP1 is a member of the dynamin superfamily of GTPases, which is required for mitochondrial fission through interacting with mitochondrial outer membrane-anchored proteins 40, 41. It also plays a crucial role in the selective removal of mitochondria through mitophagy 42. Although excessive overexpression of DRP1 leads to mitochondrial fragmentation and cell death, DRP1 gene KO mice is embryonic lethal, implicating the necessity of DRP1 in maintaining homeostasis 43. DRP1 loss in neurons, hepatocytes or cardiomyocytes decreased mitophagy and led to neurodegeneration, liver damage or lethal heart failure, respectively 38, 43-47, while downregulation of DRP1 attenuated fibroblast activation and renal fibrosis, suggesting the organ-specific role of DRP1 48. Moreover, a review has concluded that the differential effects of Mdivi1 on cell survival or death could be attributed to the different durations of treatment 49. Most *in vitro* studies on the toxic effect of Mdivi1 were performed for more than 16 hours, whereas studies reporting the protective effect of Mdivi1 were conducted for a much shorter duration (≤ 8 hours, usually ≤ 1 hour), suggesting that chronic inhibition of Drp1 might be detrimental to cell function and survival 47, 49. In the absence of DRP1, mitophagy is inhibited and mitophagy intermediates, containing p62, LC3 and mitochondrial protein, are accumulated in the hepatocytes, neurons and cardiomyocytes of the corresponding liver-, brain- and heart-specific DRP1 knockout mice, respectively 38, 44, 45. Of note, mitochondrial enlargement and the increased expressions of p62, LC3 and mitochondrial protein in DRP1 knockout mice are significantly restored after simultaneous loss of DRP1 and OPA1, suggesting that DRP1-mediated mitochondrial division leads to repression of p62, LC3 and mitochondrial protein 38. The present study demonstrated that overexpression of DRP1 restored IS-inhibited mitophagy and mitigated IEC injury. Therefore, targeted approaches that modulate mitophagy during kidney-gut injury might be a promising therapeutic strategy, including maintaining the physiologically coordinated DRP1 expression 31.

As a protein-bound uremic toxin, IS cannot directly regulate the transcription of DRP1. After bioinformatics analysis, screening and validation, we found that IRF1 was indispensable for IS-mediated DRP1 suppression *in vitro* and *in vivo*. IRF1 is a transcriptional factor containing a repression domain within the C-terminal portion, negatively regulating the transcription of its target genes 50. In this study, luciferase assay, ChIP and mutation analysis suggested that IRF1 inhibited DRP1 transcription via directly binding to its promoter region (-508 ~ -497, CACAGTGAAACC). In CKD mice and patients, increased IRF1 and decreased DRP1 expression were observed. IS-induced DRP1 repression and mitophagy blockade were also reversed in IRF1^-/-^ mice, further validating the regulation of DRP1 expression by IRF1. According to a previous study 51, IRF1 was lowly expressed in the brain, heart, kidney, testis and colon, while DRP1 was highly expressed in the brain, testis, kidney, colon and heart. Moreover, IRF1 was a tumor suppressor gene, while DRP1 promoted cancer survival 50, 52. These reports indicate a negative correlation between IRF1 and DRP1. Of interest, mitochondrial fission stabilizes the crucial downstream IRF1, while DRP1 contributes to mitochondrial fission 36. Therefore, we hypothesize that the IRF1-DRP1 axis may form a feedback loop, delicately regulating mitochondrial function to achieve a physiological balance. This hypothesis needs more experimental confirmation in the future study. In addition, as to the upstream of IRF1, previous reports have identified IRF1 as the downstream gene of AhR, which is the only proven endogenous receptor of IS 27, 28. Suppression of AhR attenuated IS-induced IRF1 upregulation, indicating an intermediary role of AhR between IS and IRF1.

In conclusion, IS, as a protein-bound uremic toxin and metabolite of gut flora, plays a crucial role in inducing CKD-associated intestinal barrier injury. This effect of IS is mainly due to its inhibition of mitophagy in IECs. Intervention strategies to control IS level or target IRF1-DRP1 axis-mediated mitophagy impairment may alleviate CKD-associated intestinal barrier injury.

## Materials and Methods

Detailed materials and methods can be found in Supplementary Materials.

### Human samples

A total of 12 CKD patients at stage 5 (predialysis, aged 22-46 years) and 12 healthy controls (aged 24-44 years) were enrolled in this study (Department of Nephrology of Xinqiao Hospital, Chongqing, China). Three pieces of human intestinal tissues were collected using colonoscopy, and the serum was used for IS concentration analysis. Informed written consent was obtained from all participants. All procedures involving human subjects were approved by the Ethics Committee of Xinqiao Hospital of the Army Medical University (No. 2018-006-01) and in accordance with the guidelines in the Declaration of Helsinki.

### Animal study

IRF1 knockout (IRF1^-/-^) mice were purchased from Jackson Laboratory (Bar Harbor, ME, USA). To construct a CKD model, 8-week-old male Balb/c mice received electrocoagulation of right 2/3 renal cortex first and left total nephrectomy two weeks later, as previously described 16. CKD mice were fed a diet containing 5% AST-120 (Kremezin, Kure ha Chemical Industry, Tokyo, Japan) for 8 weeks. WT C57BL/6J and IRF1^-/-^ mice were intraperitoneally injected with IS (100 mg/kg, Sigma-Aldrich, St. Louis, MO, USA) daily for 8 weeks. Each group included at least 8 mice. All animal procedures were approved by the Committee of Ethics on Animal Experiments of the Army Medical University.

### Cell culture

Human colon epithelial cells (Caco2) and small intestinal epithelial cells (HIEC) were obtained from the American Type Culture Collection of USA (Manassas, VA, USA). Primary colon epithelial cells were isolated and cultured as previously described 53, 54.

### 16s rRNA sequencing

Fresh fecal samples of sham and CKD mice were collected for 16s rRNA sequencing and analysis using Illumina (HiSeq 2500) plateform (Illumina, Inc, San Diego, CA), as previously described 55.

### High pressure liquid chromatography (HPLC)

Blood samples of patients and mice were centrifuged for 10 minutes (min) at 3000 rpm. Serum IS was measured using HPLC as previously described 16.

### Enzyme-linked immunosorbent assay (ELISA)

Serum TNF-α, IL-1β and IL-6 in mice were measured using the corresponding ELISA kits (Boster Biological Technology, Wuhan, China) according to the manufacturer's protocol.

### *In vivo* intestinal permeability assay

Mice were gavaged with 400 μg/g bodyweight FITC-dextran (4 kDa, Sigma-aldrich), and intestinal permeability was assessed as previously reported 56.

### Transepithelial electrical resistance (TER) determination

Cells were seeded on Millicell Hanging Cell Culture Inserts (Millipore, Billerica, MA, USA) for TER determination using an epithelial voltohmmeter Millicell ERS-2 (Merck Millipore, Billerica, MA, USA).

### Reverse transcription and quantitative PCR (qPCR)

Total RNA was extracted using Trizol reagent (Invitrogen, Carlsbad, CA, USA), and qPCR was performed by using SYBR Green qPCR kit (Takara, Dalian, China), as previously described 57. The mouse and human primers for qPCR are listed in Tables S2 and S3.

### Western blot analysis

Total protein was extracted using a cell lysis buffer containing 50 mM Tris (pH 7.4), 150 mM NaCl, 1% Triton X-100, 1% sodium deoxycholate, 0.1% SDS (Beyotime, Shanghai, China) supplemented with each protease and phosphatase inhibitor cocktail tablet (Roche Diagnostics GmbH, Mannheim, Germany) per 10 ml solution. Western blot was performed as previously described 57. Primary antibodies against IRF1 (sc-514544x) and β-actin (sc-47778) were purchased from Santa Cruz Biotechnology (Dallas, TX, USA). The antibodies against P62 (5114), p-mTOR (5536), t-mTOR (2983), p-p70S6K (9234), p-4EBP1 (2855), PINK1 (6946) and Parkin (4211) were obtained from Cell Signaling Technology (CST, Danvers, MA, USA). The antibodies against DRP1 (ab56788), MFN1 (ab57602), MFN2 (ab50838), OPA1 (ab90857), FIS1 (ab71498), AhR (ab2769), COXIV (ab14744), PGC1α (ab54481) and TFAM (ab131607) were purchased from Abcam (Cambridge, MA, USA). LC3 antibody (L7543) was purchased from Sigma-Aldrich.

### ATP measurement

ATP level was measured using a luminescent ATP determination kit (A22066, Invitrogen, Carlsbad, CA, USA) containing 0.5 mM D-luciferin, 1.25 µg/mL firefly luciferase, 25 mM Tricine buffer, 5 mM MgSO_4_, 100 µM EDTA, 1 mM DTT and 5 mM ATP solution, and normalized to protein content, according to the manufacturer's protocol.

### ROS detection

Caco2 cells were incubated with CM-H2DCF-DA for 20 min, and analyzed using an Accuri C6 flow cytometer (BD Biosciences, San Jose, CA, USA).

### Mitochondrial membrane potential analysis

Cells were incubated with JC-1, and imaged using a laser scanning confocal microscope (Zeiss, LSM780, Germany) or harvested for C6 flow cytometry analysis (BD Biosciences, San Jose, CA, USA).

### Transmission electron microscopy

Cells and intestinal tissues were imaged by TEM (JEM-1400PLUS, Japan) as previously described 33.

### Immunofluorescence

Cells or intestinal tissues were incubated with antibodies, and then treated with Cy3 or FITC-conjugated secondary antibody and DAPI for laser scanning confocal microscopy (Zesis) observation.

### GFP-LC3 puncta detection, mito-tracker staining and mCherry-GFP-LC3 assay

Cells were transfected with GFP-LC3 plasmids (Beyotime) or mCherry-GFP-LC3 adenovirus (Hanbio Co.LTD, Shanghai, China), according to the manufacturer's protocol. Then, cells were stained with mito-tracker Red CMXRos (Thermo Fisher Scientific, Waltham, MA, USA) and examined with a laser scanning confocal microscope (Zesis).

### Overexpression and downregulation of target genes

The constructed overexpression plasmids or siRNAs (primers are shown in supplementary methods) were separately transfected into Caco2 cells using Lipofectamine 2000 (Invitrogen).

### Construction of reporter plasmids, point mutation and dual-luciferase reporter assay

The recombinant plasmids (the primers are listed in Table S4) and pGL3-basic were co-transfected with pRL-TK vector (Promega, Madison, WI, USA) into Caco2 cells for luciferase activity assay using a dual-luciferase reporter assay system (Promega).

### Chromatin immunoprecipitation (ChIP) assay

ChIP was performed using a ChIP kit (Millipore) as previously described 57. Briefly, the sonicated DNA was immunoprecipitated with an anti-IRF1 antibody (Santa Cruz Biotechnology), and then amplified by PCR and qPCR using primers which are listed in Table S3.

### Statistical analysis

Data are presented as mean ± SEM unless otherwise stated. Comparisons between two groups were analyzed by two-tailed unpaired Student's *t* test. Comparisons among multiple groups were tested by one-way analysis of variance (ANOVA). Statistical analyses were performed using GraphPad Prism 8. *P* < 0.05 was considered statistically significant.

## Supplementary Material

Supplementary figures and tables.

## Abbreviations

CKD: chronic kidney disease
IS: Indoxyl sulfate
rRNA: ribosomal RNA
DRP1: dynamin-related protein 1
IRF1: Interferon regulatory factor 1
Rapa: Rapamycin
TEM: transmission electron microscope
E. coli: Escherichia coli
TER: transepithelial electrical resistance
AVs: autophagic vacuoles
GFP: Green fluorescent protein
SQSTM1/P62: sequestosome 1
Baf: Bafilomycin A1
FIS1: fission 1
MFN1: mitofusin 1
MFN2: mitofusin 2
OPA1: optic atrophy 1
YY1: Yin-Yang 1
AhR: aryl hydrocarbon receptor
WT: wild type
KO: knockout
HE: hematoxylin eosin
qPCR: quantitative PCR
ChIP: Chromatin immunoprecipitation
