# Supplementary material for: Indoxyl sulfate induces intestinal barrier injury through IRF1-DRP1 axis-mediated mitophagy impairment
Source: Theranostics. 2020 Jun 5;10(16):7384–400. doi: 10.7150/thno.45455 (PMC7330852; doi:10.7150/thno.45455)
Supplement: Supplementary file 1 — Supplementary figures and tables. [file thnov10p7384s1.pdf]

Supplementary Material

Supplementary Figures and Figure Legends

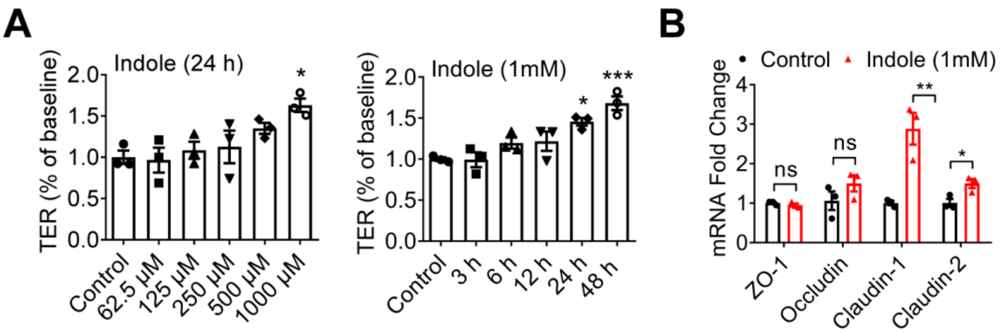

Figure S1. Indole does not disrupt intestinal barrier.

(A) Caco2 cells were seeded on Millicell Hanging Cell Culture Inserts in 24-well plates and cultured for 14 days and treated with various concentrations of Indole for 24 hours or 1 mM Indole for different durations for determination of transepithelial electrical resistance (TER). **(B)** qPCR analysis of the expressions of tight junction-related genes in Caco2 cells treated with 1 mM Indole for 24 hours. Data are shown as mean ± SEM and were analyzed by one-way ANOVA (**A**) or two-tailed unpaired Student's *t* test (**B**). n=3. ns: no significance. * $P<0.05$, ** $P<0.01$, *** $P<0.001$.

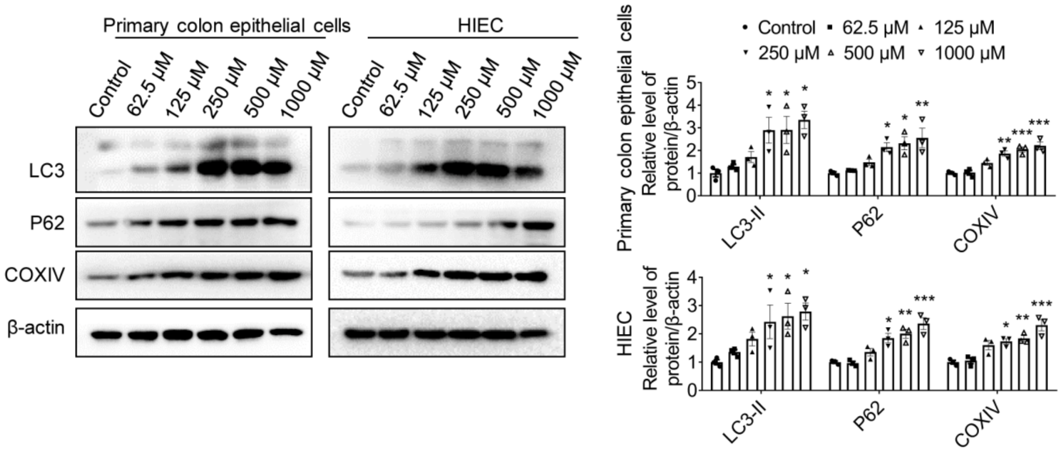

Figure S2. Indoxyl sulfate inhibits mitophagy in HIEC and primary colon epithelial cells.

Both HIEC and primary colon epithelial cells were treated with various concentrations of indoxyl sulfate for 24 hours, and then harvested for Western blot analysis to detect the expressions of LC3, P62 and COXIV. Data are shown as mean ± SEM and were analyzed by one-way ANOVA. n=3. * *P*<0.05, ** *P*<0.01, *** *P*<0.001.

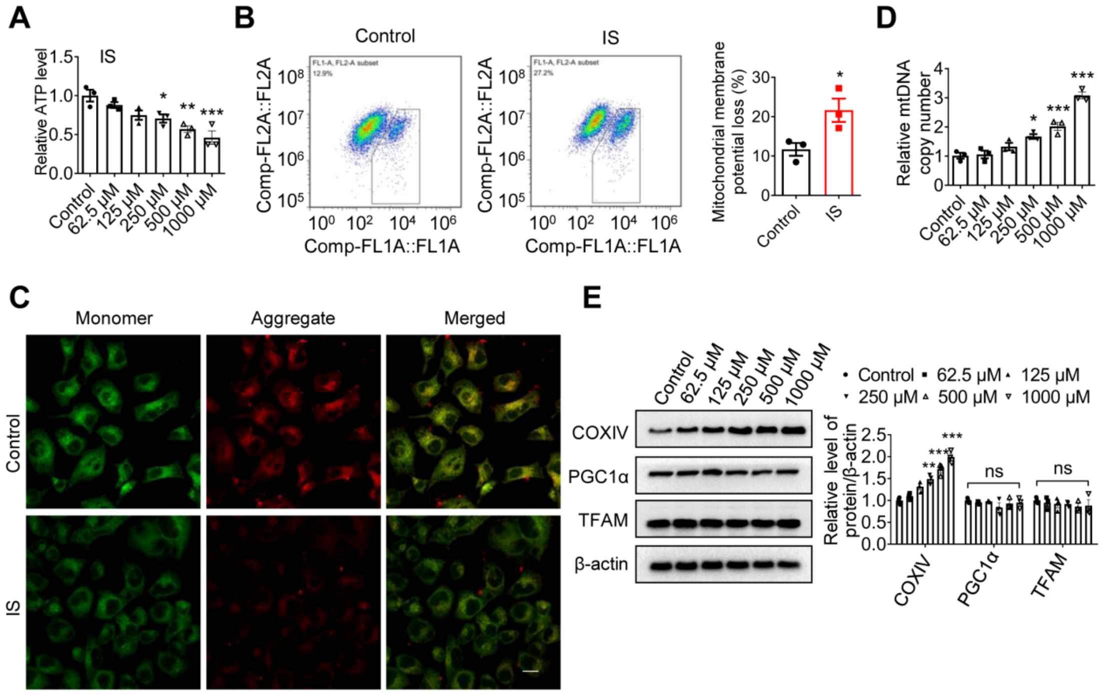

Figure S3. Mitochondrial dysfunction was induced by indoxyl sulfate.

(A) Caco2 cells were treated with various doses of IS for 24 hours. ATP levels were measured using an ATP determination kit and normalized to protein content. **(B, C)** Caco2 cells were treated with control or 500 μM IS for 24 hours and stained with JC-1 to detect mitochondrial membrane potential loss by flow cytometry **(B)** and confocal microscopy **(C)**. **(D)** qPCR analysis of mitochondrial DNA copy number in cells in **(A)**. **(E)** Western blot analysis of COXIV, PGC1α and TFAM expression in cells in **(A)**. The gray scale of bands was quantified using ImageJ software. Scale bar, 10 μm. Data are shown as mean ± SEM and were analyzed by one-way ANOVA (**A, D, E**) or two-tailed unpaired Student's *t* test (**B**). n=3. ns: no significance. * P<0.05, ** P<0.01, *** P<0.001.

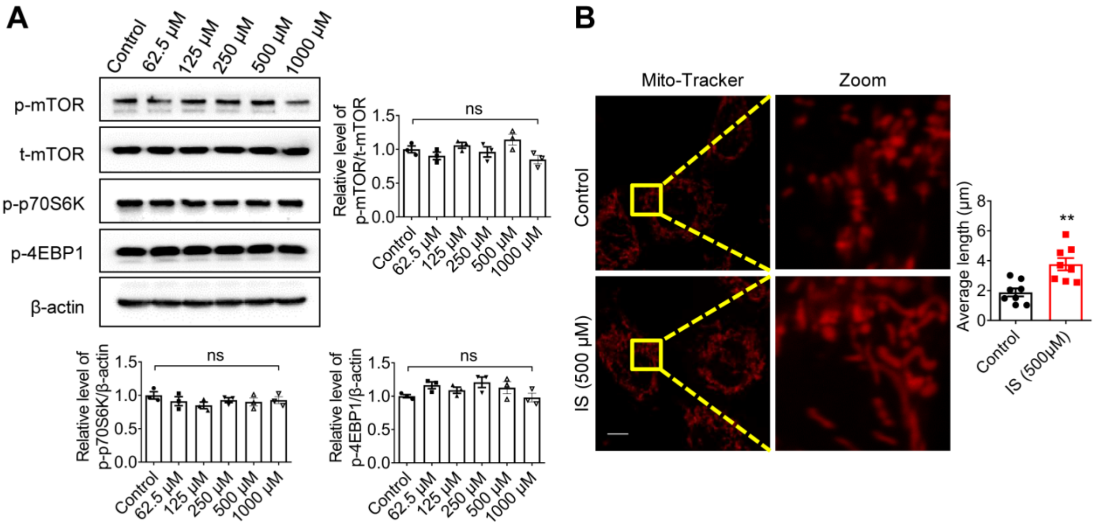

Figure S4. mTOR signaling pathway was not affected by indoxyl sulfate, but mitochondrial fusion was induced by IS in Caco2 cells.

(A) Caco2 cells were treated with various concentrations of IS for 24 hours, and then harvested for protein extraction and subsequent Western blot analysis to detect the mTOR signaling pathway. **(B)** Caco2 cells were treated with control or 500 μM IS for 24 hours and stained with 0.2 μM mito-tracker at 37 °C for 30 min to detect mitochondrial morphology by confocal microscopy. Scale bar, 10 μm. Data are shown as mean ± SEM and were analyzed by one-way ANOVA (**A**, n=3) or two-tailed unpaired Student's *t* test (**B**, n=8). ns: no significance. ** $P<0.01$.

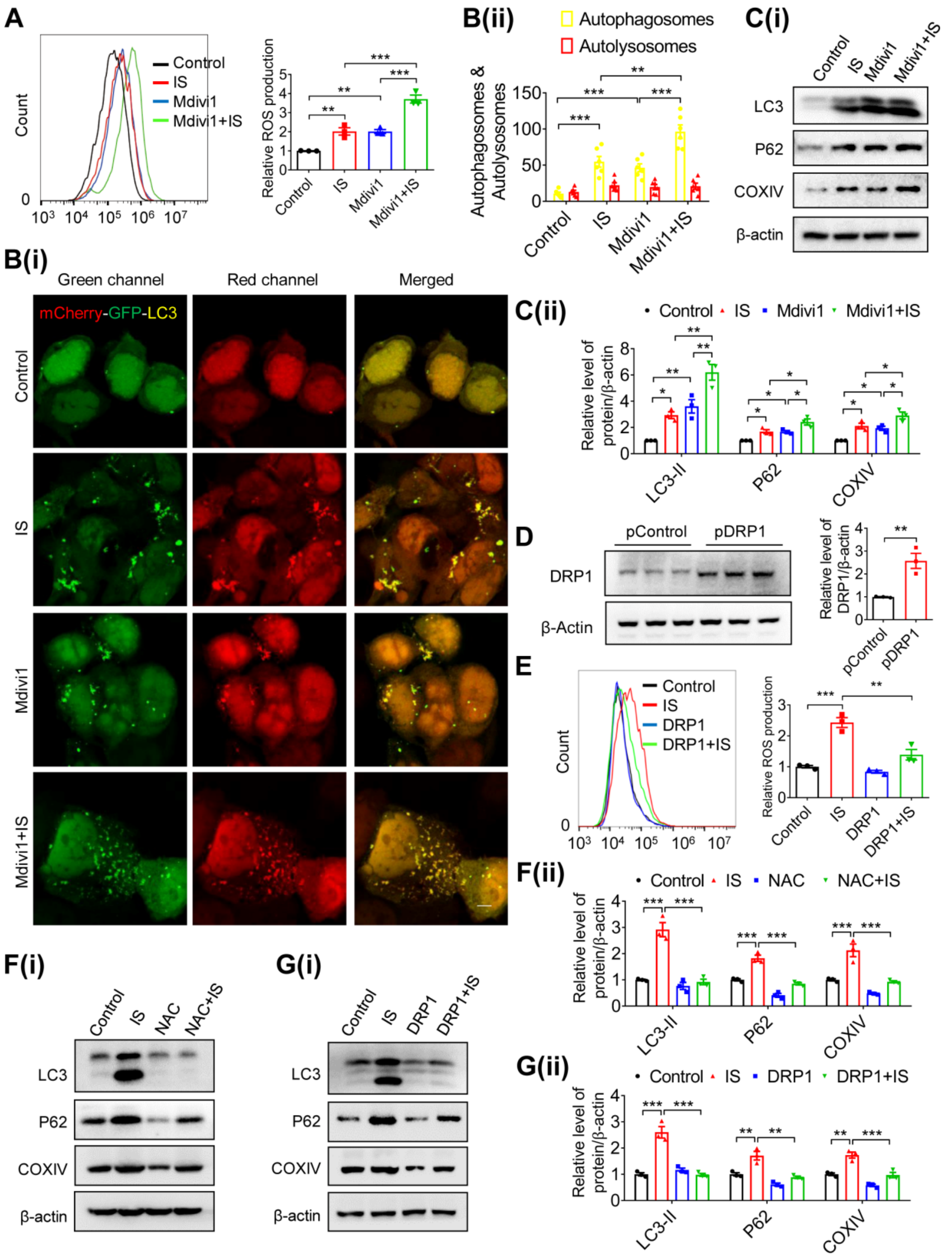

Figure S5. Indoxyl sulfate-induced ROS production and mitophagy inhibition were aggravated by Mdivi1 while mitigated by DRP1 overexpression.

(A) Caco2 cells were treated with control or 500 µM IS in the presence or absence of 25 µM Mdivi1 for 24 hours for ROS detection by flow cytometry. **(B)** Caco2 cells were transfected with mCherry-GFP-LC3, and then treated with control or 500 µM IS

in the presence or absence of 25 μM Mdivi1 for 24 hours for confocal microscopy and ImageJ analysis. Scale bar, 10 μm. **(C)** Western blot analysis of the expressions of LC3, P62 and COXIV in cells in **(A)**. **(D)** Western blot analysis of DRP1 expression in Caco2 cells transfected with vector control or DRP1 overexpression plasmids. **(E)** Caco2 cells were transfected with DRP1 overexpression plasmids or vector control and then treated with control or IS for 24 hours for ROS detection by flow cytometry. **(F, G)** Western blot analysis of LC3, P62 and COXIV expression in Caco2 cells treated with control or IS in the absence or presence of NAC or DRP1 overexpression plasmids. The gray scale of bands was quantified using ImageJ software. Data are shown as mean ± SEM and were analyzed by one-way ANOVA (**A-C, E-G**) or two-tailed unpaired Student's *t* test (**D**). n=3. * $P<0.05$, ** $P<0.01$, *** $P<0.001$.

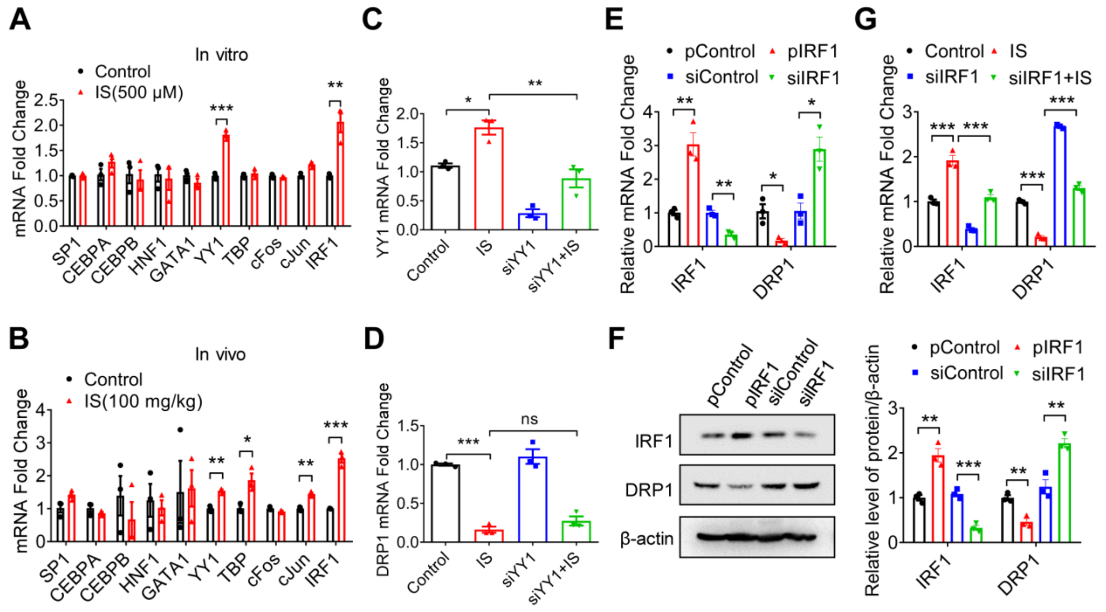

Figure S6. Screening of transcriptional factors in DRP1 promoter region.

(A, B) qPCR analysis of 10 putative transcriptional factors in DRP1 promoter region in Caco2 cells treated with control or 500 μM IS for 24 hours **(A)** and intestinal tissues from mice treated with control or 100 mg/kg IS for 8 weeks **(B)**. **(C, D)** Caco2 cells were transfected with siRNA against YY1 (siYY1) or control siRNA using Lipofectamine 2000, and then treated with control or 500 μM IS for another 24 hours. Total RNA was extracted for qPCR analysis to detect YY1 and DRP1 expression. **(E, F)** qPCR and Western blot analysis of IRF1 and DRP1 expression in Caco2 cells transfected with vector control (pControl), IRF1 overexpression plasmids (pIRF1), control siRNA (siControl) or siRNA against IRF1 (siIRF1), respectively. **(G)** Caco2 cells were transfected with siControl or siIRF1, and then treated with control or IS for 24 hours. Cells were harvested for qPCR analysis to detect the expression of IRF1 and DRP1. The gray scale of bands was quantified using ImageJ software. Data are shown as mean ± SEM and were analyzed by two-tailed unpaired Student's *t* test (**A, B**) or one-way ANOVA (**C-G**). n=3. ns: no significance. * P<0.05, ** P<0.01, ***

$P<0.001$.

Supplementary Materials and Methods

Human samples

A total of 12 CKD patients at stage 5 (predialysis, aged 22-46 years) and 12 healthy controls (aged 24-44 years) were enrolled in this study (Department of Nephrology of Xinqiao Hospital, Chongqing, China). Three pieces of intestinal tissues were collected by colonoscopy: (1) frozen in liquid nitrogen for qPCR and Western blot analyses; (2) perfused with 4% paraformaldehyde at room temperature for hematoxylin-eosin (HE) staining and immunofluorescence; (3) fixed in 2.5% glutaraldehyde at 4°C for TEM observation. Their serum was used for IS concentration analysis. Informed written consent was obtained from all participants. All procedures involving human subjects were approved by the Ethics Committee of Xinqiao Hospital of the Army Medical University (No. 2018-006-01) and in accordance with the guidelines in the Declaration of Helsinki.

Animal study

IRF1 knockout ($IRF1^{-/-}$) mice were purchased from Jackson Laboratory (Bar Harbor, ME, USA). C57BL/6J and Balb/c mice were purchased from Beijing Huafukang Bioscience (Beijing, China). To construct a CKD model, 8-week-old male Balb/c mice received electrocoagulation of right 2/3 renal cortex first and left total nephrectomy two weeks later, as previously described [1]. Laparotomy without damaging the kidney served as sham mice. CKD mice were fed a diet containing 5% AST-120 (Kremezin, Kure ha Chemical Industry, Tokyo, Japan) for 8 weeks. WT C57BL/6J and $IRF1^{-/-}$ mice were intraperitoneally injected with IS (100 mg/kg,

Sigma-Aldrich, St. Louis, MO, USA) daily for 8 weeks. Each group included at least 8 mice. All mice were randomly divided into various groups and blinded to the investigators. Mice were euthanized by carbon dioxide inhalation, and the serum and intestinal tissues were collected. Macroscopic score of the disease grade was assessed as previously described [2]. All animal procedures were approved by the Committee of Ethics on Animal Experiments of the Army Medical University.

16s rRNA sequencing and bioinformatic analysis

Fresh fecal samples of sham and CKD mice were collected for 16s rRNA sequencing and analysis by Beijing Genomics Institute (Beijing, China) as previously described [3]. Briefly, the quality of DNA extracts was determined using NanoDrop ND-2000 (Thermo Fisher, Waltham, MA, USA), and DNA integrity was examined by agarose gel electrophoresis. The qualified DNA was used to construct a library. The V4 region of 16s rRNA gene was obtained using PCR, and the products were purified for sequencing on the Illumina (HiSeq 2500) plateform (Illumina, Inc, San Diego, CA). The numbers of tag and operational taxonomic unit (OTU) were generated from the above samples. Based on the OUT abundance, Venn diagram was drawn with VennDiagram of software R (v3.1.1). Principal Component Analysis (PCA) was performed with the relative OTU abundance value using package “ade4” of software R (v3.1.1). A dot represents each sample, and different colors represent different groups. The alpha diversity comparison was analyzed by Wilcoxon Rank-Sum test, and the plotbox was drawn with software R (v3.1.1). Heatmap was generated using the package “gplots” of software R (v3.1.1) and the distance algorithm is “euclidean",

the clustering method is “complete”.

High pressure liquid chromatography (HPLC)

Blood samples of patients and mice were centrifuged for 10 minutes (min) at 3000 rpm. Serum level of IS was measured using HPLC as previously described [1].

Enzyme-linked immunosorbent assay (ELISA)

Serum levels of TNF-α, IL-1β and IL-6 in mice were determined using the corresponding ELISA kits (Boster Biological Technology, Wuhan, China) according to the manufacturer’s protocol.

***In vivo* intestinal permeability assay**

Intestinal permeability was assessed as previously reported [4]. Briefly, mice were gavaged with 400 μg/g bodyweight FITC-dextran (4 kDa, Sigma-aldrich). After 3 hours, plasma were collected for fluorescence at excitation/emission wavelength of 490/530 nm using an Infinite M200 Microplate Reader (Tecan, Manedorf, Switzerland).

Cell culture

Human colon epithelial cells (Caco2) and human small intestinal epithelial cells (HIEC) were obtained from the American Type Culture Collection of USA (Manassas, VA, USA) and cultured in Dulbecco's Modified Eagle's Medium (DMEM, Gibco, Grand Island, NY, USA) supplemented with 10% fetal bovine serum (FBS, Gibco) and 1% streptomycin/penicillin (Beyotime, Shanghai, China) at 37 °C in humidified atmosphere (5% CO_2). Primary colon epithelial cells were isolated and cultured as previously described with minor modifications [5, 6]. Briefly, the freshly excised

colons were washed with ice-cold Hanks's balanced salt solution (HBSS) (Gibco, BRL, California, CA, USA), sliced into small pieces, and then gently rocked in isolation buffer containing EDTA at 4 °C for 30 minutes under a sterile condition. After settling down, the supernatants were removed. The individual colon epithelial cells were passed through a 70 μm cell strainer (BD Falcon, Lincoln Park, NJ, USA), and then cultured in the 24 well plate pretreated with Matrigel in high-glucose DMEM medium (Gibco) supplemented with 10% FBS (Gibco) and 1% streptomycin/penicillin (Beyotime) for indicated time at 37 °C in humidified atmosphere (5% CO_2). Three independent experiments were performed, unless otherwise stated.

Trans-epithelial electrical resistance (TER) determination

Totally, 1×10^5 Caco2 cells were seeded on Millicell Hanging Cell Culture Inserts (Millipore, Billerica, MA, USA) in triplicates, which were placed in 24-well plates. After 14 days, cells were treated with IS (Sigma-Aldrich) or Indole (Sigma-Aldrich) at various concentrations for different durations. Then, TER was determined using an epithelial voltohmmeter Millicell ERS-2 (Merck Millipore, Billerica, MA, USA).

Reverse transcription and quantitative PCR (qPCR)

Total RNA was extracted using Trizol reagent (Invitrogen, Carlsbad, CA, USA) and reversely transcribed with a reverse-transcript kit (Promega, Madison, WI, USA). qPCR was performed with SYBR Green qPCR kit (Takara, Dalian, China), as previously described [7]. Total DNA was extracted from Caco2 cells using a DNA extraction kit (Takara), and mitochondrial DNA (mtDNA) copy number was analyzed

using qPCR to detect mitochondrially encoded cytochrome c oxidase II (MT-CO2), taking glyceraldehyde-3-phosphate dehydrogenase (GAPDH) as an internal control. The mouse and human primers for qPCR are listed in Tables S2 and S3.

Western blot analysis

Total protein was extracted using a cell lysis buffer containing 50 mM Tris (pH 7.4), 150 mM NaCl, 1% Triton X-100, 1% sodium deoxycholate, 0.1% SDS (Beyotime) supplemented with each protease and phosphatase inhibitor cocktail tablet (Roche Diagnostics GmbH, Mannheim, Germany) per 10 ml solution . The protein concentration was measured with a BCA kit (Beyotime).Western blot analysis was performed as previously described [7]. Briefly, the proteins were separated with 12% SDS-PAGE, transferred into a PVDF membrane, and incubated with primary antibody at 4 °C overnight. Primary antibodies against IRF1 (sc-514544x) and β-actin (sc-47778) were purchased from Santa Cruz Biotechnology (Dallas, TX, USA). The antibodies against P62 (5114), p-mTOR (5536), t-mTOR (2983), p-p70S6K (9234), p-4EBP1 (2855), PINK1 (6946) and Parkin (4211) were obtained from Cell Signaling Technology (CST, Danvers, MA, USA). The antibodies against DRP1 (ab56788), MFN1 (ab57602), MFN2 (ab50838), OPA1 (ab90857), FIS1 (ab71498), AhR (ab2769), COXIV (ab14744), PGC1α (ab54481) and TFAM (ab131607) were purchased from Abcam (Cambridge, MA, USA). LC3 antibody (L7543) was purchased from Sigma-Aldrich. The diluted concentration of all antibodies was 1:1000, except for LC3 (1:3000). Then, the membranes were incubated with horse radish peroxidase (HRP)-conjugated secondary antibodies. Blots were analyzed using

Quantity One software (BioRad, Hercules, CA, USA). The gray scale of bands was quantified using ImageJ software (NIH, Bethesda, MD, USA).

ATP measurement

ATP level (nmol) was measured using a luminescent ATP determination kit (Invitrogen) containing 0.5 mM D-luciferin, 1.25 μg/mL firefly luciferase, 25 mM Tricine buffer, 5 mM $MgSO_4$, 100 μM EDTA, 1 mM DTT and 5 mM ATP solution, and normalized to protein content (mg) determined by BCA assay (Beyotime), according to the manufacturer's protocol.

ROS detection

After treatment with IS or Indole, Caco2 cells were incubated with CM-H2DCF-DA at 37 °C for 20 min, and analyzed using the Accuri C6 flow cytometer (BD Biosciences, San Jose, CA, USA).

Mitochondrial membrane potential analysis

After treatment, Caco2 cells were incubated with JC-1 at 37 °C for 20 min, washed with JC-1 buffer and placed in culture medium on ice. Cells were imaged using a laser scanning confocal microscope (Zeiss, LSM780, Germany) or harvested for C6 flow cytometry analysis (BD Biosciences).

Transmission electron microscopy

Cells and intestinal tissues were harvested and imaged using a TEM (JEM-1400PLUS, Japan) as previously described [8].

Immunofluorescence

After treatment, cells were fixed with 4% paraformaldehyde for 20 min, incubated

with triton for 20 min and stained with anti-AhR antibody (Abcam, 1:50) at 4 °C overnight. Intestinal tissues of mice and human were incubated with anti-IRF1 (CST, 8478, 1:50, rabbit) and anti-DRP1 (Abcam, 1:50, mouse) antibodies at 4 °C overnight. Then, they were incubated with Cy3-conjugated goat anti-rabbit antibody or FITC-conjugated goat anti-mouse antibody in the dark for 1 hour and DAPI for 3 min. Cells or tissue sections were observed using a laser scanning confocal microscope (Zesis).

GFP-LC3 puncta determination and mito-tracker staining

To assess autophagy, Caco2 cells were seeded on coverslips (NEST, Wuxi, China) and transfected with GFP-LC3 plasmids (Beyotime) using Lipofectamine 2000 (Invitrogen), according to the manufacturer's protocol. Then, cells were treated with control or IS for another 24 h. Caco2 cells, with or without GFP-LC3 transfection, were treated with control or IS for 24 h and stained with mito-tracker Red CMXRos (M7512, Thermo Fisher Scientific, Waltham, MA, USA) at 37 °C for 10 min. Cells were examined using a laser scanning confocal microscope (Zesis).

mCherry-GFP-LC3 assay

To evaluate autophagic flux, Caco2 cells were seeded on coverslips (NEST) and transfected with mCherry-GFP-LC3 plasmids (Beyotime) using Lipofectamine 2000 (Invitrogen), according to the manufacturer's protocol. After 24 h, cells were treated with control, IS, Rapa or Baf for another 24 h, and then examined using a laser scanning confocal microscope (Zesis). Images were analyzed with the ImageJ software.

Overexpression and downregulation of target genes

PCR was performed to obtain full-length cDNA of target genes. After digestion, recombination, transformation and culture of the positive monoclones, the bacteria were identified by Beijing Genomics Institute (Beijing, China). The restriction enzyme for DRP1 and IRF1 was BamHI and EcoRI, respectively, and the vector was pcDNA3.1. Primers of IRF1 and DRP1 cDNA are listed as follows, and the cutting sites of restriction enzymes BamHI and EcoRI are underlined. IRF1, Forward: 5′-GCTCGGATCCATGCCCATCACTCGGATGCG-3′, Reverse: 5′-TGCAGAATTCCTACGGTGCACAGGGAATGG-3′. DRP1, Forward: 5′-GCTCGGATCCATGGAGGCGCTAATTCCTGT-3′, Reverse: 5′-TGCAGAATTCTCACCAAAGATGAGTCTCCC-3′. siRNA targeted to human IRF1, YY1 and AhR were synthesized by Sangon (Shanghai, China) and the corresponding primers are listed as follows. Negative control, Forward: 5′-UUCUCCGAACGUGUCACGUTT-3′, Reverse: 5′-ACGUGACACGUUCGGAGAATT-3′. siIRF1, Forward: 5′-CCAGAUCCCAUGGAAGCAUTT-3′, Reverse: 5′-AUGCUUCCAUGGGAUCUGGTT-3′. siYY1, Forward: 5′-AAGAUGAUGCUCCAAGAACTT-3′, Reverse: 5′-GUUCUUGGAGCAUCAUCUUTT-3′. siAhR, Forward: 5′-GCAACAAAGGAUCGGGAUATT-3′, Reverse: 5′-UAUCCCGAUCCUUUGUUGCTT-3′.

Overexpression plasmids (IRF1 and DRP1) and siRNAs (against IRF1 or AhR) were

separately transfected into Caco2 cells using Lipofectamine 2000 (Invitrogen) in OptiMEM (Hyclone, Logan, Utah, USA), according to the manufacturer's protocol. Then, cells were treated with control or IS for another 24 h and harvested for subsequent analysis.

Construction of reporter plasmids and point mutation

Putative IRF1 binding sites in DRP1 promoter region are listed in Table S1. Truncated DRP1 promoter fragments were amplified by PCR using genomic DNA of Caco2 cell (the primers are listed in Table S4). The PCR products including DRP1-2000 (-2000 ~ +200), DRP1-1000 (-1000 ~ +200), DRP1-800 (-800 ~ +200), DRP1-600 (-600 ~ +200), DRP1-300 (-300 ~ +200) and DRP1-150 (-150 ~ +200) were separately cloned into pGL3-basic after digestion by KpnI and Nhel, and the recombinant plasmids were named as pGL3-DRP1-P1, pGL3-DRP1-P2, pGL3-DRP1-P3, pGL3-DRP1-P4, pGL3-DRP1-P5 and pGL3-DRP1-P6. The predicted binding sites in pGL3-DRP1-P4 (-508 ~ -497, CACAGTGAAACC) were mutated into pGL3-DRP1-M4 (-508 ~ -497, CGCGGTGGAGCC) using the MutanBEST kit (Takara). Negative control plasmid pGL3-PGC1α-P3 (-728 ~ -717, CAAAATAACACC) was mutated into pGL3-PGC1α-M3 (-728 ~ -717, CGAGATAGCGCC). The sequences were identified by Beijing Genomics Institute.

Dual-luciferase reporter assay

The recombinant plasmids and pGL3-basic were co-transfected with pRL-TK vector (Promega) into Caco2 cells separately using Lipofectamine 2000 (Invitrogen), according to the manufacturer's protocol. Cells were treated with control or IS for

another 24 h. Luciferase activity was detected using a Dual-luciferase reporter assay system (Promega). The firefly activity was normalized against Renilla activity.

Chromatin immunoprecipitation (ChIP) assay

ChIP was performed using a ChIP kit (Millipore) as previously described [7]. Briefly, Caco2 cells were treated with control or IS for 24, fixed with 1% formaldehyde, lyzed in SDS lysis buffer, sonicated to shear DNA, and immunoprecipitated with 2 μg antibody against IRF1 (Santa Cruz Biotechnology), taking IgG as a negative control. The precipitated DNA was amplified by PCR and qPCR with the primers (-584 ~ -401) that cover the IRF1 binding sites (-508 ~ -497) in pGL3-PGC1α-P3. Primers (-1136 ~ -1020) without IRF1 binding sites served as a negative control, while the total DNA (Input) served as a positive control. The primers for ChIP are listed in Table S3.

Statistical analysis

Data were presented as mean ± SEM unless otherwise stated. Comparisons between two groups were analyzed by two-tailed unpaired Student's *t* test. Comparisons among multiple groups were tested by one-way analysis of variance (ANOVA). Statistical analyses were performed using GraphPad Prism 8. $P < 0.05$ was considered statistically significant.

References

1. Yang K, Wang C, Nie L, Zhao X, Gu J, Guan X, et al. Klotho Protects Against Indoxyl Sulphate-Induced Myocardial Hypertrophy. J Am Soc Nephrol. 2015; 26: 2434-46.

2. Jang SE, Hyam SR, Jeong JJ, Han MJ, Kim DH. Penta-O-galloyl-beta-D-glucose

ameliorates inflammation by inhibiting MyD88/NF-kappaB and MyD88/MAPK signalling pathways. Br J Pharmacol. 2013; 170: 1078-91.

3. Zhao L, Huang Y, Lu L, Yang W, Huang T, Lin Z, et al. Saturated long-chain fatty acid-producing bacteria contribute to enhanced colonic motility in rats. Microbiome. 2018; 6: 107.

4. Lee JS, Tato CM, Joyce-Shaikh B, Gulen MF, Cayatte C, Chen Y, et al. Interleukin-23-Independent IL-17 Production Regulates Intestinal Epithelial Permeability. Immunity. 2015; 43: 727-38.

5. Kim MH, Kang SG, Park JH, Yanagisawa M, Kim CH. Short-chain fatty acids activate GPR41 and GPR43 on intestinal epithelial cells to promote inflammatory responses in mice. Gastroenterology. 2013; 145: 396-406 e1-10.

6. Sato T, Clevers H. Primary mouse small intestinal epithelial cell cultures. Methods Mol Biol. 2013; 945: 319-28.

7. Huang Y, Zhou J, Huang Y, He J, Wang Y, Yang C, et al. SARI, a novel target gene of glucocorticoid receptor, plays an important role in dexamethasone-mediated killing of B lymphoma cells. Cancer Lett. 2016; 373: 57-66.

8. Huang Y, Zhou J, Luo S, Wang Y, He J, Luo P, et al. Identification of a fluorescent small-molecule enhancer for therapeutic autophagy in colorectal cancer by targeting mitochondrial protein translocase TIM44. Gut. 2018; 67: 307-19.

Supplementary Tables

Table S1. The putative binding sequences of IRF1 in DRP1 promoter region

Name	Score	Relative Score	Start	End	Strand	Predicted binding sites
IRF1	9.5784	0.828322	-282	-271	+	GGAAGTGACACT
IRF1	11.9915	0.882112	-508	-497	+	CACAGTGAAACC
IRF1	8.7531	0.809926	-728	-717	+	CAAAATAACACC
IRF1	8.7438	0.809719	-915	-904	-	CATAGTGAGACC
IRF1	8.67381	0.808159	-1342	-1331	+	CATGGTGAAACC

Table S2. The primer sets for mouse qPCR

Gene (mouse)	Primers	Product size
β-actin	Forward: 5′-TGTTACCAACTGGGACGACA-3′<br>Reverse: 5′-GGGGTGTTGAAGGTCTCAAA-3′	165 bp
ZO-1	Forward: 5′-ACCTCTGCAGCAATAAAGCAG-3′<br>Reverse: 5′-GAAATCGTGCTGATGTGCCA-3′	230 bp
Occludin	Forward: 5′-TTCAGGTGAATGGGTCACCG-3′<br>Reverse: 5′-AGATAAGCGAACCTGCCGAG-3′	162 bp
Connexin43	Forward: 5′-GGTGTGGATGGACCTTATGCT-3′<br>Reverse: 5′-GGAAGACACAAAGGTGGGACA-3′	149 bp
Claudin-1	Forward:5′-TGCACATGCCTTCAACTGTTCT-3′<br>Reverse: 5′-ACAAAACCTTGCTCTGCTGAAG-3′	158 bp
Claudin-2	Forward: 5′-ATGCCTTCTTGAGCCTGCTT-3′<br>Reverse: 5′-AAGGCCTAGGATGTAGCCCA-3′	218 bp
DRP1	Forward: 5′-GCCTCAGATCGTCGTAGTGG-3′<br>Reverse: 5′-TTTTCCATGTGGCAGGGTCA-3′	194 bp
SP1	Forward: 5′-GTCAGCGTCCGCGTTTTTC-3′<br>Reverse: 5′-CGCTACCCCCATTATTGCCA-3′	163 bp
CEBPA	Forward: 5′-TTCGGGTCGCTGGATCTCTA-3′<br>Reverse: 5′-TCAAGGAGAAACCACCACGG-3′	164 bp
CEBPB	Forward: 5′-CGCCTTATAAACCTCCCGCT-3′<br>Reverse: 5′-TGGCCACTTCCATGGGTCTA-3′	167 bp

HNF1	Forward: 5′-GTGCCCACAGAGCTTGACTA-3′<br>Reverse: 5′-TAGAAACCATGGCTCCGCTG-3′	168 bp
GATA1	Forward: 5′-GGATCACCCTGAACTCGTCA-3′<br>Reverse: 5′-CAGAATCCACAAACTGGGGC-3′	144 bp
YY1	Forward: 5′-GCCCTCATAAAGGCTGCACA-3′<br>Reverse: 5′-TCTCAACGAACGCTTTGCCA-3′	117 bp
TBP	Forward: 5′-GTGCCAGATACATTCCGCCT-3′<br>Reverse: 5′-AGCTGCGTTTTTGTGCAGAG-3′	113 bp
cFos	Forward: 5′-TACTACCATTCCCCAGCCGA-3′<br>Reverse: 5′-GCTGTCACCGTGGGGATAAA-3′	113 bp
cJun	Forward: 5′-GGGAGCATTTGGAGAGTCCC-3′<br>Reverse: 5′-TTTGCAAAAGTTCGCTCCCG-3′	182 bp
IRF1	Forward: 5′-CTTCGTCGAGGTAGGACGTG-3′<br>Reverse: 5′-CTTTGCTGCAGGAGCGATTC-3′	214 bp

Table S3. The primer sets for human qPCR

Gene (human)	Primers	Product size
β-actin	Forward: 5′-GTGAAGGTGACAGCAGTCGGTT-3′<br>Reverse: 5′-GAAGTGGGGTGGCTTTTAGGA-3′	157 bp
ZO-1	Forward: 5′-AGCCATTCCCGAAGGAGTTG-3′<br>Reverse: 5′-ATCACAGTGTGGTAAGCGCA-3′	175 bp
Occludin	Forward: 5′-GGTCTAGGACGCAGCAGATT-3′<br>Reverse: 5′-GCCTGGATGACATGGCTGAT-3′	98 bp
Claudin-1	Forward: 5′-CTGGGAGGTGCCCTACTTTG-3′<br>Reverse: 5′-ACACGTAGTCTTTCCCGCTG-3′	106 bp
Claudin-2	Forward: 5′-TCATGGGATCCTACGGGACT-3′<br>Reverse: 5′-TCCTTGTGGCAAGAGGTTGG-3′	200 bp
DRP1	Forward: 5′-AGAAAATGGGGTGGAAGCAGA-3′<br>Reverse: 5′-AGGCACCTTGGTCATTCCTG-3′	220 bp
FIS1	Forward: 5′-GTCCAAGAGCACGCAGTTTG-3′<br>Reverse: 5′-ATGCCTTTACGGATGTCATCATT-3′	75 bp
MFN1	Forward: 5′-TGGCTAAGAAGGCGATTACTGC-3′<br>Reverse: 5′-TCTCCGAGATAGCACCTCACC-3′	185 bp
MFN2	Forward: 5′-CTCTCGATGCAACTCTATCGTC-3′<br>Reverse: 5′-TCCTGTACGTGTCTTCAAGGAA-3′	173 bp
OPA1	Forward: 5′-TGTGAGGTCTGCCAGTCTTTA-3′<br>Reverse: 5′-TGTCCTTAATTGGGGTCGTTG-3′	141 bp

SP1	Forward: 5′-CCCTTGAGCTTGTCCCTCAG-3′<br>Reverse: 5′-TGAAAAGGCACCACCACCAT-3′	136 bp
CEBPA	Forward: 5′-CCAGAAAGCTAGGTCGTGGG-3′<br>Reverse: 5′-TCCTAGGCAATGCTGAAGGC-3′	153 bp
CEBPB	Forward: 5′-TTTGTCCAAACCAACCGCAC-3′<br>Reverse: 5′-GCATCAACTTCGAAACCGGC-3′	154 bp
HNF1	Forward: 5′-CCTGTGCAGAGCCATGTGAC-3′<br>Reverse: 5′-TCTGAGGTGAAGACCTGCTTG-3′	203 bp
GATA1	Forward: 5′-CTACACCAGGTGAACCGGC-3′<br>Reverse: 5′-CCACCACCATAAAGCCACCA-3′	151 bp
YY1	Forward: 5′-GTGCCCCTTCGATGGTTGTA-3′<br>Reverse: 5′-GGAAGATGCTTCCCGTGGTC-3′	137 bp
TBP	Forward: 5′-CATTATCAACGCGCGCCAG-3′<br>Reverse: 5′-CCCTGGGTCACTGCAAAGAT-3′	122 bp
cFos	Forward: 5′-AACCGCCACGATGATGTTCT-3′<br>Reverse: 5′-TCTGCGGGTGAGTGGTAGTA-3′	114 bp
cJun	Forward: 5′-GAGCTGGAGCGCCTGATAAT-3′<br>Reverse: 5′-CCCTCCTGCTCATCTGTCAC-3′	104 bp
IRF1	Forward: 5′-CATGCCCTCCACCTCTGAAG-3′<br>Reverse: 5′-CCATCCACGTTTGTTGGCTG-3′	111 bp
MT-CO2	Forward: 5′-CAAACCTACGCCAAAATCCA-3′<br>Reverse: 5′-GAAATGAATGAGCCTACAGA-3′	164 bp

GAPDH	Forward: 5′-TGACAACAGCCTCAAGAT-3′<br>Reverse: 5′-GAGTCCTTCC ACGATACC-3′	195 bp
DRP1 (-584 ~ -401)	Forward: 5′- GCCTGTAATCCCAACACTTT-3′<br>Reverse: 5′- GTTCACGCCATTCTCCTG-3′	184 bp
DRP1 (-1136 ~ -1020)	Forward: 5′- AGAACGGTATGAGCAAAGA-3′<br>Reverse: 5′- GGAAGAGCTGGGCATTTA-3′	117 bp

The primers of *DRP1* (-584 ~ -401) and *DRP1* (-1136 ~ -1020) were used for ChIP assays.

Table S4. The primer sets for PCR amplification of the human *DRP1* promoter region

Fragment	Primers (The cutting sites of restriction enzymes KpnI and Nhel are underlined)
-2000 ~ +200	Forward: 5′- GATAGGTACCGCCTCACTCTGTCACTTAGG-3′<br>Reverse: 5'- CCGGGCTAGCCTGGAGCTTGTTTATGACAG-3'
-1000 ~ +200	Forward: 5'- GATAGGTACCCTAAGATTTTTTCATGAAC-3'<br>Reverse: 5'- CCGGGCTAGCCTGGAGCTTGTTTATGACAG-3'
-800 ~ +200	Forward: 5'- GATAGGTACCGTTTGTTTGTTTGTTTACAAAAC-3'<br>Reverse: 5'- CCGGGCTAGCCTGGAGCTTGTTTATGACAG-3'
-600 ~ +200	Forward: 5'- GATAGGTACCAAGTCACACTGCTCACGCCTG-3'<br>Reverse: 5'- CCGGGCTAGCCTGGAGCTTGTTTATGACAG-3'
-300 ~ +200	Forward: 5'- GATAGGTACCATAAAAAATAAATAAAAGGAAG-3'<br>Reverse: 5'- CCGGGCTAGCCTGGAGCTTGTTTATGACAG-3'
-150 ~ +200	Forward:5'- GATAGGTACCCCTAGCCTTTGACTAGAGCC-3'<br>Reverse: 5'- CCGGGCTAGCCTGGAGCTTGTTTATGACAG-3'

Restriction enzymes: KpnI and Nhel, Vector: pGL3-basic.

Table S5. Macroscopic injury score of mice and human

Sham	0	0	0	1	1	1	1	0				
CKD	4	4	3	2	5	4	5	3				
Control	0	0	1	1	0	0	1	0				
IS	2	3	3	4	2	3	2	4				
Healthy donors	0	0	0	1	1	0	0	0	1	0	0	0
CKD patients	4	5	4	2	5	2	3	4	3	2	2	1
Sham	1	0	1	1	0	0	1	0				
CKD	3	4	4	5	2	3	4	5				
CKD+AST-120	1	2	4	1	1	0	2	1				
Control	1	0	0	1	1	0	1	0				
IS	3	5	2	3	4	2	5	3				
IRF1$^{-/-}$	0	0	1	1	0	0	1	1				
IRF1$^{-/-}$+IS	2	4	2	1	0	1	2	1				
